# A toolkit for the identification of NEAT1_2/paraspeckle modulators

**DOI:** 10.1093/nar/gkac771

**Published:** 2022-09-13

**Authors:** Haiyan An, Karen T Elvers, Jason A Gillespie, Kimberley Jones, John R Atack, Olivera Grubisha, Tatyana A Shelkovnikova

**Affiliations:** Medicines Discovery Institute, School of Biosciences, Cardiff University, Cardiff CF10 3AT, UK; Medicines Discovery Institute, School of Biosciences, Cardiff University, Cardiff CF10 3AT, UK; Medicines Discovery Institute, School of Biosciences, Cardiff University, Cardiff CF10 3AT, UK; Medicines Discovery Institute, School of Biosciences, Cardiff University, Cardiff CF10 3AT, UK; Medicines Discovery Institute, School of Biosciences, Cardiff University, Cardiff CF10 3AT, UK; Medicines Discovery Institute, School of Biosciences, Cardiff University, Cardiff CF10 3AT, UK; Medicines Discovery Institute, School of Biosciences, Cardiff University, Cardiff CF10 3AT, UK; Sheffield Institute for Translational Neuroscience, Department of Neuroscience, University of Sheffield, Sheffield S10 2HQ, UK

## Abstract

Paraspeckles are ribonucleoprotein granules assembled by NEAT1_2 lncRNA, an isoform of Nuclear Paraspeckle Assembly Transcript 1 (NEAT1). Dysregulation of NEAT1_2/paraspeckles has been linked to multiple human diseases making them an attractive drug target. However currently NEAT1_2/paraspeckle-focused translational research and drug discovery are hindered by a limited toolkit. To fill this gap, we developed and validated a set of tools for the identification of NEAT1_2 binders and modulators comprised of biochemical and cell-based assays. The NEAT1_2 triple helix stability element was utilized as the target in the biochemical assays, and the cellular assay (‘ParaQuant’) was based on high-content imaging of NEAT1_2 in fixed cells. As a proof of principle, these assays were used to screen a 1,200-compound FDA-approved drug library and a 170-compound kinase inhibitor library and to confirm the screening hits. The assays are simple to establish, use only commercially-available reagents and are scalable for higher throughput. In particular, ParaQuant is a cost-efficient assay suitable for any cells growing in adherent culture and amenable to multiplexing. Using ParaQuant, we identified dual PI3K/mTOR inhibitors as potent negative modulators of paraspeckles. The tools we describe herein should boost paraspeckle studies and help guide the search, validation and optimization of NEAT1_2/paraspeckle-targeted small molecules.

## INTRODUCTION

Once referred to as ‘junk DNA’, the vast majority of human genome is now known to produce a diverse spectrum of non-coding RNA species, which do not serve as templates for protein synthesis yet play key regulatory roles in multiple cellular processes (1). Non-coding transcripts that are longer than 200 nucleotides are classed as long non-coding RNAs (lncRNAs); they often exhibit spatial- and temporal-specific expression patterns indicative of their tight regulation and hence functionality (2). More than 200 human diseases, most prominently multiple types of cancer, neurological and cardiovascular conditions, are attributable to mutations affecting lncRNAs or to their dysregulation via other mechanisms (3).

Nuclear Paraspeckle Assembly Transcript 1 (NEAT1) is an abundant, ubiquitously expressed nuclear-retained lncRNA. NEAT1 is one of the most studied disease-linked lncRNAs, and its modulation may provide benefit in a wide range of human conditions, from cancer to neurodegenerative diseases and viral infections. The *NEAT1* locus produces two transcripts, NEAT1_1 and NEAT1_2, and the latter transcript is essential for the assembly of nuclear ribonucleoprotein (RNP) granules paraspeckles, such that loss of NEAT1_2 expression results in paraspeckle disintegration (4). Although paraspeckles contain multiple proteins and other RNAs, NEAT1_2 serves as the only reliable marker of these structures and as such is a proxy for paraspeckles. Contribution of NEAT1_2/paraspeckles to human disease originates from a plethora of regulatory roles in gene expression, e.g. control of the availability/active pool of transcription factors or sequestration of specific mRNAs in the nucleus (5–8). They were also found to regulate histone post-translational modifications (9) and contribute to miRNA biogenesis (10). NEAT1_2 is the stress-inducible NEAT1 isoform, and murine studies demonstrated that within normal tissue, constitutive NEAT1_2 expression is restricted to the cell populations in the gut and in the reproductive system (11,12). However, accumulation of NEAT1_2 is typical for many tumours and for other pathological contexts characterized by significant cellular stress, i.e. viral infections and neurodegeneration (13).

NEAT1 dysregulation is a recurrent theme in malignant tumours. *NEAT1* gene is a hotspot for mutations in some types of cancer, and NEAT1 is overexpressed in the majority of solid tumours, including breast, prostate, lung, colorectal, and gastric cancer (14–17). NEAT1_2, but not NEAT1_1, likely plays a pivotal role in tumorigenesis, at least in some types of cancer (18,19). In particular, studies in mice suggested that NEAT1_2/paraspeckles promote tumorigenesis by increasing the survival of oncogene-targeted cells, where downregulation NEAT1_2 was sufficient to sensitize cancer cells to chemotherapy (20).

NEAT1 upregulation is a general response to viral infections. The *NEAT1* gene product was initially identified as a ‘viral-induced non-coding RNA’ (VINC) in the mouse brain (21). Multiple viruses induce NEAT1, including HIV-1, dengue, herpes simplex virus and hepatitis D virus, among others (22). NEAT1 has been associated with the response to SARS-CoV-2 (23). NEAT1_2/paraspeckles positively regulate cellular antiviral responses by sequestering the transcription factor SFPQ away from the promoters of genes it represses in basal state, such as *CXCL8* and *CCL5* (5,24). NEAT1_2/paraspeckles can be both proviral and antiviral, depending on the type and timing of infection.

The link between NEAT1 and neurological, including neurodegenerative, diseases has recently emerged. NEAT1_2 upregulation coupled with *de novo* paraspeckle assembly is one of the characteristic features for motor neurons in amyotrophic lateral sclerosis (ALS) patients (25,26). NEAT1 is also elevated in the affected brain regions in Huntington's, Parkinson's and Alzheimer's disease, frontotemporal dementia, epilepsy and traumatic brain injury, where it may play a neuroprotective and prosurvival role, although possible differential contribution of the two isoforms is yet to be clarified (27).

Given the accumulating evidence on the pathological significance of NEAT1_2 and its specific tissue expression profile, it clearly represents an attractive therapeutic target. Antisense oligonucleotide probes, such as gapmers and morpholino oligonucleotides were used, so far only as experimental tools, for NEAT1_2/paraspeckle modulation (28,29). Small molecule ligands for RNA provide a number of advantages over oligonucleotides, such as better cellular uptake and superior pharmacokinetic properties. The use of small molecules to modulate RNA, although traditionally considered challenging and limited to antiviral/antibacterial research, has recently gained more traction (30,31). It is now clear that despite the linearity of RNA molecules and limited complexity of the primary sequence, higher-order assembly motifs (e.g. hairpins, bulge loops) and points of interaction between RNAs and RNA-binding proteins can provide suitable pockets for small molecule binding (32). The breakthrough in the field was the development of two small molecule RNA splicing modulators, branaplam and risdiplam for a fatal childhood disease spinal muscular atrophy (SMA) (33,34). Both molecules were identified in a phenotypical assay validating the use of phenotypic approaches in RNA drug discovery. *In vitro* and computational approaches are also able to support RNA focused translational work (35). For instance, recently, small-molecule microarrays enabled the discovery of chemotypes binding to MALAT1 lncRNA (36), and an *in silico* study was successful in identifying small molecules binding to an iron response element in the 5′ UTR of alpha-synuclein mRNA (37).

The progress in the identification of small molecules for NEAT1_2/paraspeckle modulation is currently hindered by the lack of robust screening and validation approaches. In the current study, we developed a toolkit for the identification of NEAT1_2/paraspeckle small molecule binders, comprised of a biochemical assay and secondary biophysical/binding and cellular assays. The assays were established in two screens, using a 1200-molecule library of FDA-approved drugs and a kinase inhibitor library, yielding novel negative and positive modulators of NEAT1_2/paraspeckles and new mechanistic insights in NEAT1_2/paraspeckle regulation.

## MATERIALS AND METHODS

### *In vitro* reconstitution and characterization of NEAT1_2 triple helix (TH)

#### TH assembly

The 93-nt TH motif of NEAT1_2 was assembled using two synthetic RNA oligonucleotides (‘fragments’) f1: 5′-AGGUGUUUCUUUUACUGAGUGCAGCCC-3′ (27 nt) and f2: 5′-UGGCCGCACUCAGGUUUUGCUUUUCACCUUCCCAUCUGUGAAAGAGUGAGCAGGAAAAAGCAAAA-3′ (65 nt). Lyophilized RNA pellets were resuspended in RNase-free water to 100 μM, aliquoted and stored at −80°C. For TH reconstitution, the two oligonucleotides were mixed in equimolar ratio in a buffer of optimized composition containing 20 mM Tris–HCl pH 7.0, 100 mM KCl, 4 mM MgCl_2_ (‘folding buffer’), denatured at 90°C for 5 min and snap-cooled on ice for 10 min. 30S ribosomal A-site RNA (5′-CGGCGUCACACCUCGGGGUGAAGUCGCCG-3′) was resuspended in a buffer containing 20 mM Tris–HCl pH 7.0 and 100 mM KCl, denatured and snap-cooled as above. RNA oligonucleotides were synthesized and HPLC-purified by Eurofins.

#### Thermal melting

Melting analysis was performed using CFX384 Real-Time PCR Detection System (Bio-Rad). Thiazole green (SYBR Green I, Thermofisher) fluorescence change was used as an indicator of TH structural disruption. For each reaction, 9 μl of 1 μM RNA sample was mixed with 1 μl dye (x10) and transferred to a thermal PCR plate (Bio-Rad). Fluorescence signal was recorded over the temperature range from 65°C to 90°C (ramp rate of 0.5°C).

#### Native polyacrylamide gel electrophoresis

Hand-cast gels were prepared with 12% acrylamide (29:1 mono:bisacrylamide), 34 mM Tris–HCl pH 7.0, 66 mM HEPES pH 7.0, 0.1 mM EDTA and 4 mM MgCl_2_. Gels were pre-run for 30 min in running buffer containing 34 mM Tris–HCl, 66 mM HEPES pH 7.0, 0.1 mM EDTA and 4 mM MgCl_2_, at a constant voltage of 60 V. RNA samples (5 μM, 4 μl per well) were mixed with 1 μl of 5× RNA loading dye (running buffer containing 50% glycerol and bromophenol blue). Low Range ssRNA Ladder (New England Biolabs) was used as a molecular weight marker. The gel was run for 4 h on ice, followed by incubation in 10% acetic acid for 10 min and staining with SYBR™ Safe (Thermofisher) for 5 min. The signal was detected using ChemiDoc™ Gel Imaging System (Bio-Rad).

### Fluorescent intercalator displacement (FID) assay for NEAT1_2 TH

TWO-PRO™-1 (equivalent to TO-PRO^®^-1) was purchased from AAT Bioquest, and work solution was prepared in the folding buffer. Black 96-well plates (FluoroNunc™) containing 40 μl of TWO-PRO-1 solution per well were scanned on PHERAstar FSX Microplate Reader (BMG Labtech) to obtain the basal fluorescence reading at 485 nm/520 nm (ex/em). Equal volume of 1 μM RNA substrate was added to the dye, incubated for 5 min and scanned to record fluorescence in the presence of RNA. To test compound binding, 1.6 μl of compound stock was added to the above mixture with fluorescence reading recorded after 5 min. Compounds used for FID assay development were as follows: paromomycin (Alfa Aesar, J61274.06), chloramphenicol (Sigma, C0378-5G), G418 (Sigma, 727878001), ribocil (Cayman Chemical, 18487), LMI070 (Cayman Chemical, 26757), linezolid (Enzo LifeSciences, LKT-L3453-M100) and compound 5 (synthesized in-house) (36). For a miniaturized version of the assay, 384-well black F-bottom microplates (Greiner, 784076-25) were used, and the reaction volume was reduced (see Results) with all other parameters preserved.

### LOPAC^®^1280 library screen using NEAT1_2 FID

The Library of Pharmacologically Active Compounds (LOPAC^®1280^) was purchased from Sigma. Working plates with 500 μM compounds in DMSO (16 × 96-well plates, 80 compounds per plate, first and last column empty) were used in this screen. On the day of the screen, working plates were thawed at room temperature, vortexed and centrifuged at 1000×g for 2 min. Compounds were tested at a final concentration of 10 μM by adding 1.6 μl compound to wells containing 80 μl of RNA/TWO-PRO-1 mixture (2.0% final DMSO concentration). Paromomycin was used as a positive control at a final concentration of 500 μM. Both ‘no RNA’ (folding buffer instead of RNA) and ‘no compound’ (with DMSO added instead of compound) controls were included. The library was screened in single replicates on PHERAstar FSX Microplate Reader. Compound activity was assessed using the normalized percent displacement (NPD) metric: NPD = (FFCnegm − FFCtest)/(FFCnegm – FFCposm) × 100%, where FFC (fluorescence fold change) = fluorescence after compound addition/fluorescence after compound addition. FFCnegm is the mean FFC of negative controls; FFCposm is the mean FFC of positive controls; FFCtest is FFC of individual test compounds. Compounds with NPD value >70% were considered as hits. *Z*’ value was calculated for each plate using PHERAstar FSX MARS software. Dose–response analysis was performed in triplicates using the same parameters as in the main screen, and curve fitting and IC_50_ calculation were performed using GraphPad Prism 6.0.

### Analysis of small molecule binding to NEAT1_2 TH by grating-coupled interferometry (GCI) with waveRAPID approach

Surface-based biophysical measurements of binding kinetics were performed on a Creoptix^®^WAVE system (Creoptix, AG) using 4PCH STA WAVE sensor chips (polycarboxylate surface, streptavidin coated). Chips were conditioned with borate buffer (100 mM sodium borate pH 9.0, 1M NaCl; Xantec). RNA oligonucleotides f1 and f2 with a 5′ biotin–TEG modification were synthesized and HPLC-purified by Eurofins. NEAT1_2 TH was assembled by denaturing and snap-cooling as described above. Working solutions of NEAT1_2 TH, f1 and f2 were prepared in HBSM buffer (10 mM HEPES pH7.4, 150 mM NaCl, 0.005% Tween-20, 4 mM MgCl_2_) at the final concentration of 10 μM. TH and individual fragments were immobilized on the sensor chip by injection onto the surface at a flowrate of 10 μl/min. One channel was left blank. The final surface density for all RNAs was ∼2000 pg/mm^2^ (between 1905 and 2026 pg/mm^2^ in a representative experiment). Kinetic analyses were performed at 25°C using the waveRAPID (Repeated Analyte Pulses of Increasing Duration) and ‘intermediate binders’ settings (38). Compounds were tested consecutively, at a final concentration of 10 μM, in HBSM buffer with 1% DMSO, at a flow rate of 100 μl/min. Compounds were injected for 25 s total injection duration followed by 300 s dissociation with assay buffer. Blank injections were used for double referencing. Data analysis and visualization were performed using the WAVEcontrol software 4.0 (correction applied: X and Y offset; DMSO calibration; double referencing). Kinetic parameters were calculated using Direct Kinetics engine and one-to-one binding model.

### ParaQuant: cell culture and treatments

SH-SY5Y, HeLa and U2OS cells were purchased from ECACC repository via Sigma and maintained in DMEM/F12 supplemented with 10% foetal bovine serum and 1× Penicillin–Streptomycin–Glutamine (all from Invitrogen). Cells were plated onto 96-well CellCarrier-96 Ultra Microplates (PerkinElmer) one day before treatment at a density of 2000–8000 cells per well, depending on the cell line, in 100 μl of media. Human embryonic stem (ES)-cell derived motor neurons were differentiated as described earlier (39). Day 36 neurons were plated onto Matrigel/laminin-coated 96-well CellCarrier-96 Ultra Microplates and left to grow until Day 40. Proteasome inhibitor MG132 (Merck) was used at 6.5 μM and NaAsO_2_ (Merck)—at 0.5 mM. Compounds were added to the culture media as specified in the Results and/or figure legends (the day after plating for stable cell lines). Working plates of the Cayman Chemical Kinase Screening library (10505) were prepared by diluting 10 mM stock solutions of compounds in water to 500 μM. For the initial screen, 1 μl compound was added to the cells growing in 100 μl of media in a 96-well format. Compound validation studies were performed in a 96-well or 384-well format as described above, in ≥3 replicates (see Results/figure legends for specific experiments).

### ParaQuant: RNA fluorescent *in situ* hybridization (FISH) and immunocytochemistry

All volumes are indicated per well of a 96-well plate. All solution changes and washes were done using a 12-channel pipette and 12-channel aspirator adaptor to ensure rapid processing and consistent conditions across wells. After application of the probe, all incubations were done in the dark. Cells were washed with 1× PBS (100 μl) once, fixed with ice-cold 4% paraformaldehyde (PFA) in PBS (50 μl) for 20 min at RT and left in 75% ethanol (100 μl) overnight at 4°C. Plates were kept at 4°C up to a week. On the day of RNA-FISH and imaging, cells are washed with 100 μl of 2× SSC (Sigma, SRE0068) prepared in nuclease-free water, twice for 5 min each. Cells were incubated in 50 μl of hybridization buffer (20% formamide in 2× SSC) for 15 min. Stellaris^®^ FISH Probe Human NEAT1 Middle Segment with Quasar^®^ 570 Dye (Biosearch Technologies, SMF-2037-1) was dissolved in 100 μl of nuclease-free water and kept in −80°C. Probe was used at 10 nM final concentration in hybridization buffer (50 μl per well). Cells were incubated at 37°C for 6 h or at RT overnight. Probe was aspirated and cells were left in 100 μl of 1× PBS for 30 min at RT. For stress granule co-staining or neuronal network co-labeling, CoraLite^®^488-conjugated anti-G3BP1 (ProteinTech, CL594-66486) and Alexa^®^488-conjugated anti-betaIII-tubulin (Abcam, ab237350) antibodies were used, respectively. Cells were incubated with the fluorescently labelled antibody for 30 min in 1xPBS without detergents and washed with 1× PBS once. For nuclei visualization, cells were incubated in 100 μl of DAPI solution (50 ng/ml in 1× PBS) for 5 min at RT. DAPI solution was replaced by 100 μl 1× PBS for imaging. Plates were imaged 15 min after DAPI staining or kept at 4°C for up to a week. Plates were left to equilibrate at RT for at least 30 min before imaging.

### ParaQuant: high-content confocal imaging and quantification

Cells were imaged using 40× water objective on Operetta CLS or Opera Phenix (PerkinElmer) and analysed using Harmony 4.9 software. The following imaging parameters were used: Cy3 (NEAT1_2/paraspeckles), 600 ms, laser power 80%; DAPI (nuclei), 100 ms, laser power 50%. Three planes between −1.0 μm and 1.0 μm were scanned in each field, and maximum intensity projection images were used for analysis. Between 6 and 12 fields were typically analysed per well, with fields both in the centre and in the periphery included. The following building blocks were used for image analysis: Find nuclei: method B; Find spots: method B. Within the population ‘Nuclei’, three parameters were recorded: number of objects (nuclei); number of spots (paraspeckles) per cell − mean per well; spot area [px²] − mean per cell − mean per well. Stress granule assembly was also analysed using the spot analysis tool in the G3BP1 channel (method B, spot Area [px²] − mean per cell − mean per well). CSIRO neurite analysis module of Harmony was used for assessing the integrity of the network in motor neurons.

### Mechanistic studies of kinase inhibitor hits

Kinase inhibitor library hits for cellular validation experiments were purchased from Apexbio/Stratech.

#### Generation of NEAT1_2 knockout cells

Guide RNA (gRNA) sequences targeting *NEAT1* gene region that corresponds to the non-overlapping sequence between NEAT1_1 and NEAT1_2 transcripts were identified using Feng Zhang lab's Target Finder (http://crispr.mit.edu/). Two gRNA sequences were selected on each end of the target region to increase editing efficiency. Forward and reverse oligonucleotides Upstream1 5′-TACATCCAAAGTCGTTATGA-3′ and Upstream2 5′-AGAACTGGTATTATCCCAAG-3′; and Downstream1 5′-CCTTGTAAAGGCATAGCCAG-3′ and Downstream2 5′-CAAAACCTGAGTGCGGCCAT-3′ were annealed and cloned into pX330-U6-Chimeric_BB-CBh-hSpCas9 (pX330) vector (Addgene). Plasmids were delivered into SH-SY5Y cells by calcium phosphate transfection. Single-cell derived sub-clones were expanded and screened by PCR using 5′-ATGGGGAAGTAGTCTCGGGT-3′ and 5′-AGGATGAGGGAGGGGATAGC-3′ primers. All primers were custom-synthesized by Merck (Sigma). NEAT1_1 distribution was analysed using Stellaris® FISH Probe Human NEAT1 5′ segment with Quasar^®^ 570 Dye (Biosearch Technologies, SMF-2036-1).

#### siRNA knockdown and qRT-PCR

Silencer^®^ Select (validated) siRNAs were purchased from Thermo Scientific. HeLa cells were transfected with siRNA using Lipofectamine2000 (Thermo Scientific) and analysed after 48 h. For gene expression analysis, total RNA was extracted from cells using QIAzol from freshly lysed samples (24-well format, ∼90% confluent) with a heating step (55°C for 10 min). First-strand cDNA synthesis was performed using 500 ng of RNA with random primers (Promega) and MMLV reverse transcriptase (Promega) as per manufacturer's protocol (25 μl final reaction volume). qRT-PCR was performed using qPCRBIO SyGreen Lo-ROX with human NEAT1, MALAT1 and GAPDH primers used previously (40), on a CFX96/C1000™ qPCR system (Bio-Rad) using a default programme (×40 cycles). Samples were analysed in two or three technical repeats (20 μl volume) and expression of specific genes was determined using the 2^–ΔΔCt^ method and GAPDH for normalization. Primers used for gene knockdown validation were: RICTOR, 5′-GAGTACGAGGGCGGAATGAC-3′ and 5′-TGATACTCCCTGCAATCTGGC-3′; RPTOR, 5′-GACCTCGTGAAGGACAACGG-3′ and 5′-TTGACGATCACGGCGAGAAT-3′; PI3KCA, 5′-AGAGCCCCGAGCGTTTCTG-3′ and 5′-TTCACCTGATGATGGTCGTGG-3′; PRKDC, 5′-ATAGCGTTGTGCCCATGACC-4’ and 5′-GGGATCACTCAGGTAAGCCG-3′. Primers were designed using the NCBI Primer design tool (BLAST + Primer3); primer pair was separated by at least one intron where possible. Amplicon size was set between 90 and 120 bp with other default parameters preserved.

#### Western blotting

Total cell lysates were prepared for western blot by adding 2× Laemmli buffer directly to the wells in a 24-well plate followed by denaturation at 100°C for 5 min. SDS-PAGE and detection of proteins were carried out as described elsewhere (26). The following commercial primary antibodies were used (1:1000 dilution): FUS (rabbit polyclonal, 11570-1-AP, Proteintech); NONO (rabbit polyclonal C-terminal, Sigma); SFPQ (rabbit monoclonal, ab177149, Abcam); beta-actin (mouse monoclonal, A5441, Sigma).

#### Immunocytochemistry and fluorescent microscopy

The same antibodies as above were used for immunocytochemistry analysis of paraspeckle protein distribution. Cells were fixed as described above for RNA-FISH, permeabilized for 5 min in methanol, washed with 1xBPS and incubated in a primary antibody in 1× PBS/0.1% Tween-20/5% goat serum (1:1000 dilution) for 2 h and subsequently in secondary antibody (1:1000 dilution, Alexa^®^546 or Alexa^®^488, ThermoFisher) for 1 h. Nuclei were visualized with DAPI. Images were taken with a 100× objective on BX57 fluorescent microscope equipped with ORCA-Flash 4.0 camera (Hamamatsu) and cellSens Dimension software (Olympus) or on Opera Phenix. ParaQuant with Stellaris^®^ FISH Human MALAT1 Quasar^®^ 570 probe (Biosearch Technologies, SMF-2035-1) was used to visualize speckles, and their quantification was performed using Spot analysis on Harmony 4.9.

### Data visualization and statistics

Processed data visualization and statistical analysis were performed using GraphPad Prism 6.0 software unless indicated otherwise. Heatmaps were generated on Harmony 4.9. Mean values of replicates were compared using appropriate statistical tests. *Z*’ was calculated using in-built software add-ons or manually using the following equation: *Z*’ = 1 – 3(SD positive control + SD negative control)/|mean positive control – mean negative control|. Statistical tests used are indicated in figure legends with statistical significance denoted with asterisks: *P < 0.05, **P < 0.01, ***P < 0.001, ****P < 0.0001. *N* indicates the number of biological replicates (usually, wells in a multi-well plate). Error bars represent standard error of the mean (SEM).

## RESULTS

### *In vitro* reconstitution of the NEAT1_2 triple helix

Non-enzymatic in-solution assays using structured RNA motifs as substrates, such as fluorescent intercalator displacement (FID) assay, have been successfully applied in screening (41). For example, FID assay was used to identify small molecule modulators of survival motor neuron (SMN) splicing that act by binding to the terminal stem-loop 2 (TSL2) hairpin at an exon/intron junction (42). The assay is based on the loss of fluorescence of a RNA-bound intercalator upon displacement by a small molecule binder. FID assay does not require covalent labelling or surface immobilization, allows the analysis of interactions between RNA and chemical probes that are both tagless, and is time-/cost-efficient.

**Figure 1.**
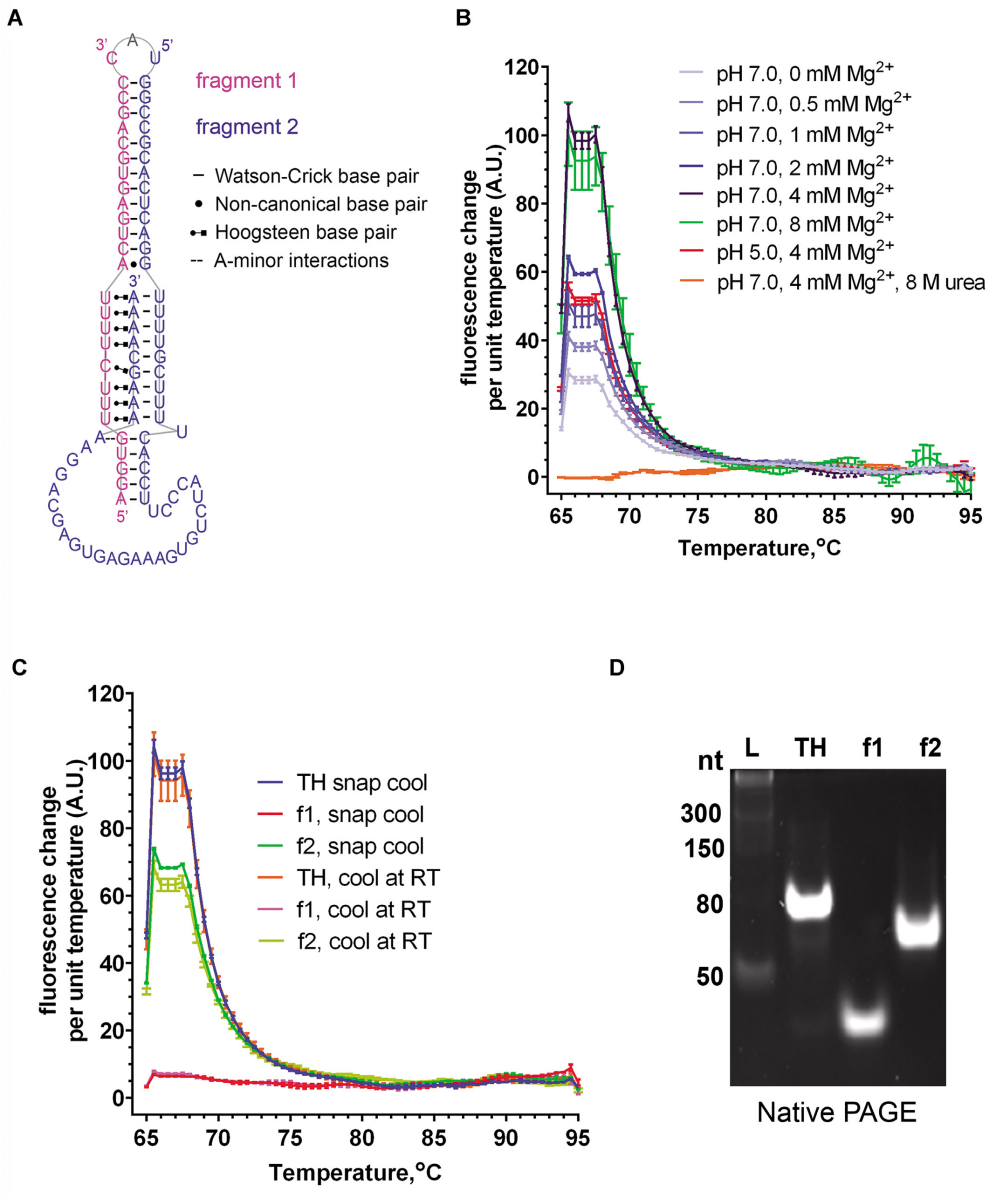
Characterisation of NEAT1_2 triple helix (TH) reconstituted *in vitro*. (**A**) Schematic representation of the NEAT1_2 TH structure. Sequences of RNA oligonucleotides ‘fragment 1’ and ‘fragment 2’ used for TH reconstitution are given in pink and blue, respectively. The apical loop adenosine excluded from the reconstituted TH is in grey. Watson–Crick base pairs are indicated with dashes, non-canonical base pairs - with dots, Hoogsteen base pairs—with Leontis-Westhof notation, and the A-minor interaction—with double dash. (**B**) Thermal melting curves for NEAT1_2 TH assembled in the ‘base’ buffer (20 mM Tris–HCl pH 7.0, 100 mM KCl) with different Mg^2+^ concentrations. Fluorescence signal of thiazole green intercalating dye added to the complex was recorded over the temperature range from 65°C to 90°C using a ramp rate of 0.5°C, and the change in the signal was plotted. Experiment was done in duplicates. (**C**) Thermal melting curves for NEAT1_2 TH and its structural RNA oligonucleotides (f1 and f2) prepared either by snap-cooling on ice and slow cooling at room temperature (RT, 25°C). Experiment was done in duplicates. (**D**) NEAT1_2 TH analysis by non-denaturing polyacrylamide electrophoresis (native PAGE). NEAT1_2 TH was reconstituted in a buffer with optimized composition (‘folding buffer’, 20 mM Tris–HCl pH 7.0, 100 mM KCl, 4 mM MgCl_2_) before loading on gel. *L* indicates RNA molecular weight marker. Representative gel is shown. Also see Supplementary Figure S1B.

Fortuitously, NEAT1_2 contains a unique structured motif—an evolutionarily conserved element for nuclear expression (ENE)—a triple helix (TH) present in the 3′ end of the molecule and critical for its stability (43–46). The TH sequence contains a genomically encoded A-rich tract flanked by two U-rich motifs, which is predicted to form stacks of four and three U•A-U triples separated by a C•G-C triplet and C-G doublet, extended by an A-minor interaction (45,47) (Figure 1A). Deletion of the TH motif in cultured cells using CRISPR dramatically decreases NEAT1_2 levels (46). The only other structurally similar TH encoded in the human genome is found in another lncRNA, Metastasis-Associated Lung Adenocarcinoma Transcript 1 (MALAT1) (44). In 2018, small molecules binding to MALAT1 TH were first identified in a screen of a library based on a RNA-binding scaffold, diphenylfuran (DPF) (48) and subsequently optimized (49). In a 2019 study, a small molecule microarray yielded compounds that bind to MALAT1 TH and are active in an organoid model (36). Small molecule probes binding to defined pockets within MALAT1 TH were also identified by *in silico* docking (50).

For NEAT1_2 TH reconstitution *in vitro*, we chose to use two RNA oligonucleotides, termed fragment 1 (27 nt) and fragment 2 (56 nt), covering the entire TH sequence except the adenosine in the apical loop (Figure 1A). The ‘break’ in the sequence was conveniently placed at the apical loop, with minimal effect expected on the TH structure. In addition, this combination allowed cost-efficient commercial synthesis which typically sets a limit of 65 nt for RNA oligonucleotides. NEAT1_2 TH was reconstituted *in vitro* by mixing the two oligonucleotides in equimolar concentration, heat denaturing and snap-annealing on ice. Mg^2+^ ions have stabilising effect on the tertiary structure of RNA and triple helices in particular, as has been shown for MALAT1 TH (36). Thermal melting analysis with thiazole green was used to assess the effect of Mg^2+^ concentration on the structure of NEAT1_2 TH in a ‘base’ buffer containing 20 mM Tris–HCl pH 7.0, 100 mM KCl (51). Two distinct peaks were observed, at 66°C and 68°C, with the height of the peaks positively correlated with Mg^2+^ concentration, up to 4 mM; further increase in Mg^2+^ did not affect the peak height. Low pH (5.0) decreased the peak height, and addition of 8M urea eliminated the peaks (Figure 1B). No difference in the peak height was observed between samples after fast annealing on ice and slower annealing at room temperature (Figure 1C). Fragment 1 alone did not show the peaks typical for NEAT1_2 TH, and the height of the peaks for fragment 2 (longer fragment) was substantially lower as compared to NEAT1_2 TH complex (Figure 1C). Base buffer containing 4 mM MgCl_2_ (termed ‘folding buffer’) was used for NEAT1_2 TH reconstitution throughout the study. The integrity of NEAT1_2 TH reconstituted under the optimal conditions was further assessed by native polyacrylamide gel electrophoresis (PAGE). NEAT1_2 TH was detected as a single band of a higher molecular weight, compared to the individual fragments, suggesting that virtually all oligonucleotides enter the complex upon annealing, forming a single conformational species (Figure 1D). We concluded that bipartite NEAT1_2 TH can be reconstituted *in vitro* as a conformationally pure complex suitable for the use in *in vitro* assays.

### Development of a fluorescent intercalator displacement assay for NEAT1_2 TH

**Figure 2.**
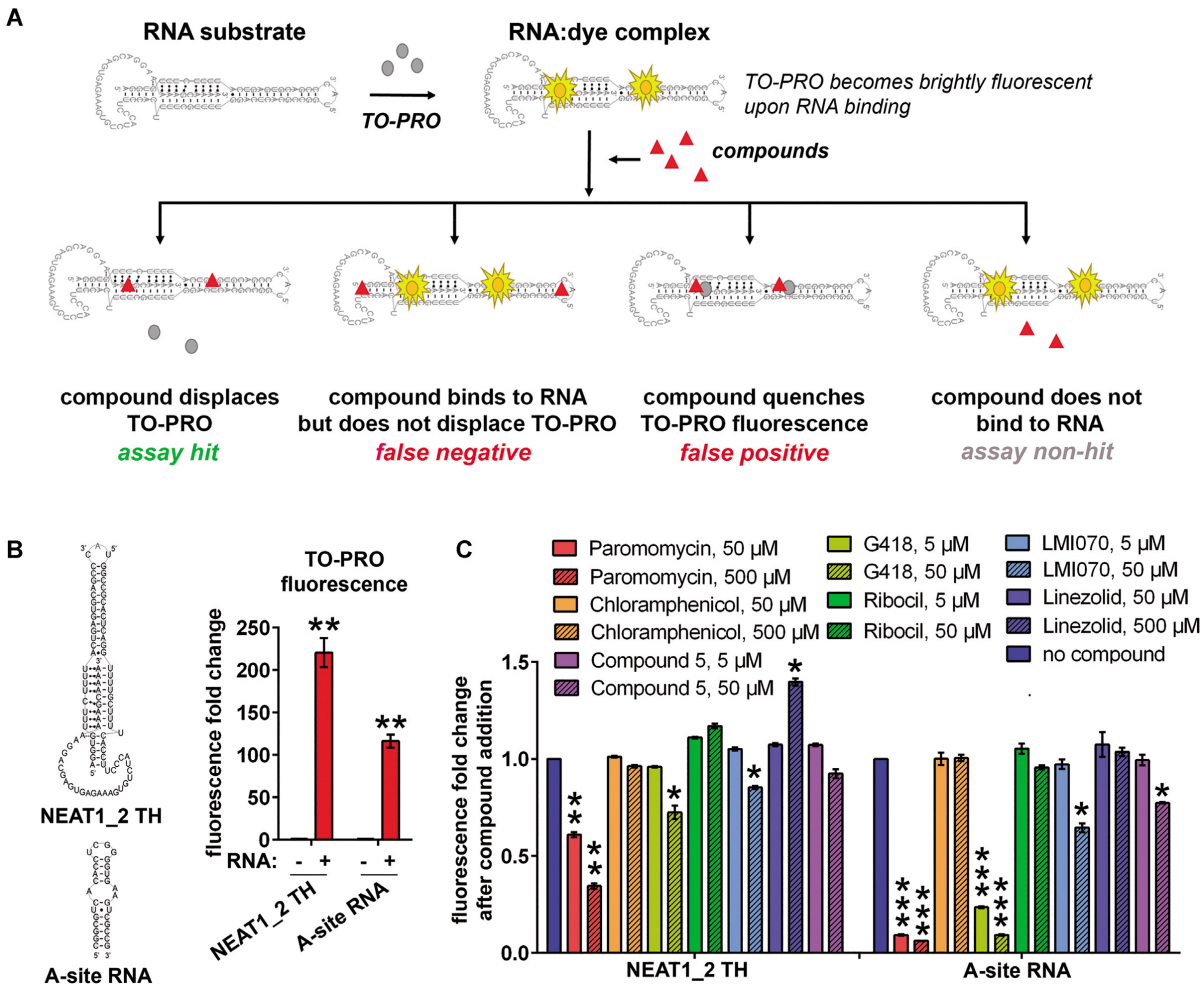
Development of a fluorescent intercalator displacement (FID) assay for NEAT1_2 TH. (**A**) FID assay principle. TO-PRO, a carbocyanine dye which has negligible fluorescence becomes brightly fluorescent upon binding RNA. Four possible outcomes upon addition of a small molecule compound to the dye/RNA complexes are shown. (**B**) TO-PRO fluorescence intensity fold-change upon its binding to NEAT1_2 TH and A-site RNA substrates. Structures of both substrates are shown for comparison. Fluorescence was analysed at 5 min after RNA addition. Final RNA concentration was 0.5 μM. *N* = 3, ** *P*< 0.01 (Mann–Whitney *U* test). (**C**) Binding of known small molecule RNA ligands to NEAT1_2 TH and A-site RNA as measured by FID. Final concentration of both RNA substrates was 0.5 μM. *N* = 3, *P< 0.05, **P< 0.01, ***P< 0.001 (one way ANOVA with Dunnett's test for multiple comparisons).

*In vitro* reconstituted NEAT1_2 TH was next used to develop a FID assay (Figure 2A). So far, FID assays have been utilized for short and simple structured RNAs such as TAR RNA (29 nt), A-site RNA (27 nt), TSL2 (19 nt) and various aptamers (41). In parallel with NEAT1_2 TH, we also tested, as a reference, A-site RNA—a short structured RNA located at the aminoacyl-tRNA site of bacterial 16S rRNA—a known target of aminoglycoside antibiotics (52).

TWO-PRO™-1 (chemical equivalent of TO-PRO^®^-1), a carbocyanine monomer nucleic acid intercalator with excitation/emission peaks of 515/531 nm and excellent fluorescence contrast between RNA-unbound and -bound states (55), was used. Mixing TWO-PRO™-1 (TO-PRO thereafter) with NEAT1_2 TH or A-site RNA in an equimolar ratio in the folding buffer led to 220.7 ± 20.0 and 116.3 ± 9.6 fold increase in the fluorescence, respectively (Figure 2B). The difference in fluorescence fold change between NEAT1_2 TH and A-site RNA is consistent with the presumed dependency of this readout on the length and number of double-stranded segments in RNA. We next tested a panel of known RNA ligands for their ability to bind to NEAT1_2 TH using this FID assay setting. Compounds were tested in two concentrations, selected based on the literature, with A-site RNA included in parallel. Paromomycin, an aminoglycoside known to bind to the A-site RNA, led to a dramatic drop in TO-PRO fluorescence in A-site RNA samples both at 50 and 500 μM (from 1.0 to 0.08 ± 0.01 on average for both concentrations). Although displacement for NEAT1_2 TH was less dramatic, a significant decrease in fluorescence was also observed with paromomycin, more pronounced for 500 μM (to 0.34 ± 0.02) as compared to 50 μM (to 0.61 ± 0.02) (Figure 2C). G418, another aminoglycoside known to bind to A-site RNA (53), decreased TO-PRO fluorescence for A-site RNA at both concentrations used, while showing only a small effect on NEAT_2 TH and only at the higher concentration (to 0.72 ± 0.04) (Figure 2C). Chloramphenicol and linezolid, both known to bind 23S rRNA but not A-site RNA (54,55), had no effect on TO-PRO fluorescence in either A-site RNA or NEAT1_2 TH samples at 50 μM; 500 μM linezolid increased the fluorescence in the NEAT1_2 TH sample (Figure 2C). Ribocil, which binds to riboflavin riboswitches (56), had no effect on either RNA (Figure 2C). Finally, LMI070 (branaplam), a splicing modulator interacting with 5′ splice site of SMN2 intron 7 (33), led to a moderate decrease in TO-PRO fluorescence for both RNAs at the higher concentration. This compound was previously shown to bind an RNA hairpin structure by NMR (33) therefore its binding to both A-site RNA and TH was expected. We also used a non-commercial small molecule ‘Compound 5’, a chemotype with 1-methyl-imadazol-2-amine scaffold recently found to specifically bind to MALAT1 TH but not NEAT1_2 TH, by NMR (36). Compound 5 did not show significant displacement in NEAT1_2 TH samples at both concentrations tested and led to a small displacement in A-site RNA samples at the higher concentration (Figure 2C). Based on these results, we concluded that FID assay performs sufficiently well for more complex RNA substrates, and that paromomycin at 500 μM can be used as a positive control in this assay.

The following four steps were taken to optimize the assay conditions in a 96-well format and convert it into a screening-ready assay. First, in order to ascertain that the variability in the quality of synthetic RNA would not affect the assay performance, we tested NEAT1_2 TH complexes assembled using RNA oligonucleotides from two independent synthesis batches, by thermal melting and native PAGE. Although variation in the height of peaks in the melting analysis was observed (Supplementary Figure S1A), NEAT1_2 TH complexes originating from two different sets of RNA oligonucleotides did not differ in native PAGE (Supplementary Figure S1B) or FID assay (Supplementary Figure S1C, D). Second, since the cost of RNA oligonucleotides accounts for >95% of the cost of assay materials, we determined the minimum reaction volumes and RNA amounts required for robust and reproducible results. Reaction volumes ranging from 40 to 120 μl were tested using the positive control paromomycin. Higher volumes did result in an improved signal window (RNA versus RNA + compound) (Supplementary Figure S2A), with the optimal volume (performance versus RNA use) established at 80 μl. Assay was found to perform well with the final RNA substrate concentrations of 0.25 μM and even 0.1 μM however the latter concentration resulted in a higher variability between the replicates (Supplementary Figure S2B). Third, the analysis of DMSO tolerance showed that DMSO concentrations of up to 5% do not significantly affect the fluorescence of the RNA/dye complex (Supplementary Figure S2C). We tested whether a drop in fluorescence at > 5% DMSO is attributable to the effect of DMSO on the NEAT1_2 TH, using thermal melting. DMSO at 10%, but not at 5%, indeed dramatically decreased the melting curve peak height (Supplementary Figure S2D). Finally, we established that NEAT1_2 TH complex was stable for at least 6 h at room temperature and after a freeze-thaw cycle (Supplementary Figure S2E), supporting the use of a complex that is batch-prepared and kept at deep-frozen (−80°C), which would help streamline the assay setup. FID assay was also successfully miniaturized to a 384-well format, with a similar performance at the reaction volumes of 20 and 10 μl and with all other assay parameters preserved (Supplementary Figure S2F).

Using paromomycin (500 μM) and DMSO (2.0%) as positive and negative assay controls, respectively, we calculated *Z*’ for 96- and 384-well formats, across three plates. The values fell in the range of 0.774–0.982 and 0.559–0.744 for the two formats, respectively, indicating the assay suitability for screening applications.

### NEAT1_2 TH FID assay validation in a 1200-compound library screen

**Figure 3.**
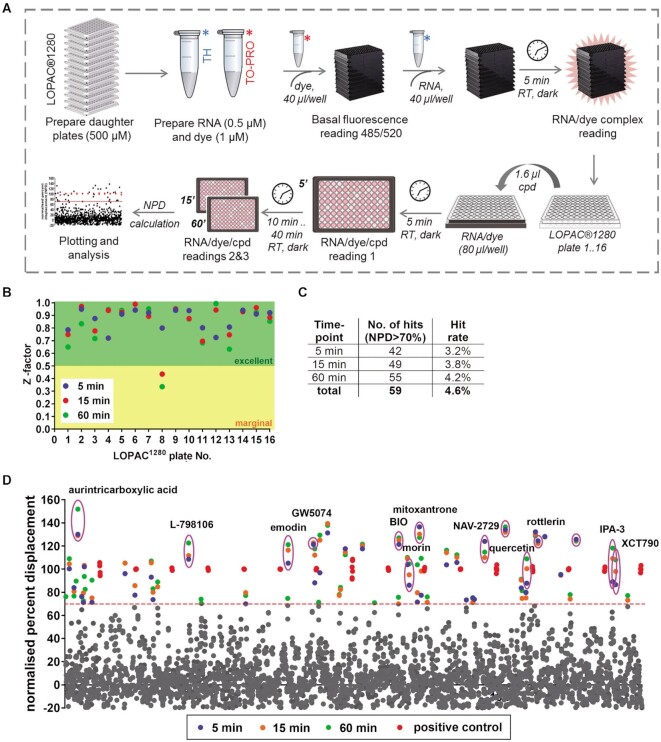
Pilot small molecule library screen with FID assay to identify NEAT1_2 TH binders. (**A**) Workflow for the LOPAC^®1280^ library screening. Final RNA concentration was 0.25 μM. LOPAC^®1280^ compounds were tested at a final concentration of 10 μM in single replicates. (**B**) Assay quality accessed using the Z’ metric across 16 LOPAC^®1280^ plates. (**C**) LOPAC^®1280^ screen hit rates. (**D**) Normalized percent displacement (NPD) values for LOPAC^®1280^ compounds in this screen. For the hits, 5-, 15- and 60-min data points are given in blue, orange and green, respectively. Red data points correspond to the positive assay control (500 μM paromomycin). Red line indicates the threshold used to identify hits (70% NPD). Data points for the compounds taken into validation studies are circled.

Having optimized NEAT1_2 TH FID assay conditions, we proceeded to a prototypical screen of a small molecule library, to validate this assay. LOPAC^®^^1280^ comprised of FDA-approved drugs and drug-like small molecules was used. The library was screened in a 96-well format at the final concentration of 10 μM in single replicates, taking advantage of the assay robustness and minimal variability between the replicates (Figure 2; Supplementary Figure S2). The reaction volume of 80 μl and final RNA concentration of 0.25 μM were used as the optimal performance/cost-efficiency combination. Workflow for the screen is shown in Figure 3A. Paromomycin (500 μM) and DMSO (2.0%) were used as positive and negative controls respectively; a ‘no RNA’ control containing only TO-PRO was also included on each plate. Fluorescence intensity readings were taken at 5, 15 and 60 min time-points after compound addition, in order to identify both ‘fast’ and ‘slow’ binders. The primary assay readout was the normalized percent displacement (NPD), where NPD of positive and negative controls were set as 100% and 0%, respectively. The average signal-to-background ratio was 4.22 ± 0.15 with a coefficient of variation of 5.9% for the signal and 13.7% for the background. *Z*’ values ranged between 0.7 and 0.9 across 16 plates (0.86 ± 0.03), confirming excellent assay quality (Figure 3B). Compounds with NPD > 70% were considered hits. After 5, 15 and 60 min incubation, 42, 49 and 55 compounds were recorded as hits (hit rate 3.2%, 3.8% and 4.2%, respectively), and 59 hits in total were identified, 20 of which had an NPD >100% (Figure 3C, D, Supplementary Figure S3A, Table S1). Several compounds appeared negative initially but qualified as hits following longer incubation, whereas several others were recorded as hits at the two early time-points only.

**Figure 4.**
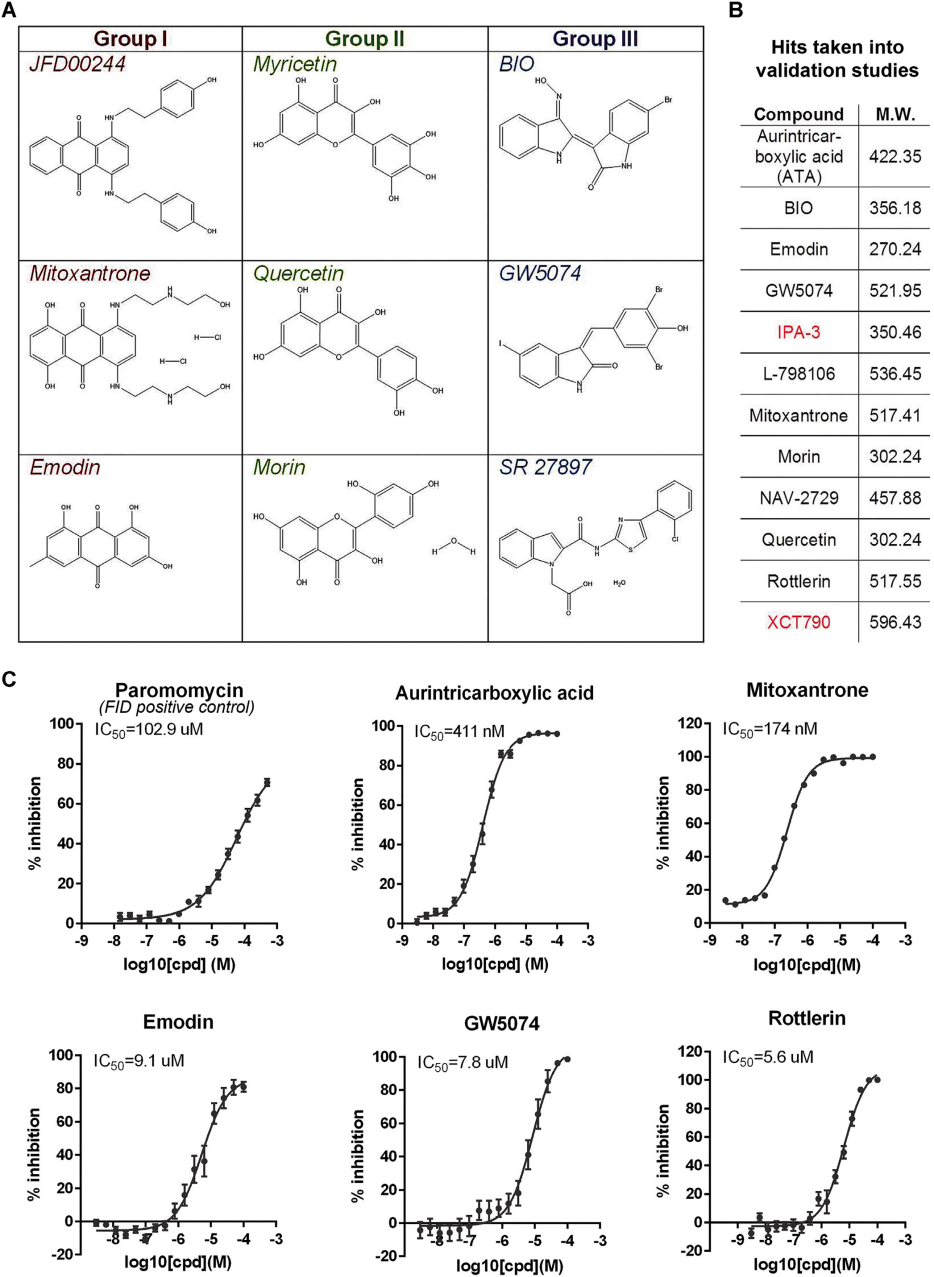
Compound chemistry grouping and dose–response analysis for LOPAC^®1280^ hits identified using the NEAT1_2 TH FID assay. (**A**) Structures of representative molecules for the three chemotype groups identified within the NEAT1_2 TH LOPAC^®1280^ hit-set. (**B**) Hits taken into validation studies and used for the secondary assay development, with their molecular weights indicated. Compounds in red failed in the concentration response studies. (**C**) Dose–response curves and IC_50_ values for selected hits determined with the primary FID assay. Paromomycin was the positive FID assay control. *N* = 3. See also Supplementary Figure S4.

Analysis of the full list of hits (all time-points, 59 compounds) showed that although the hit-set was diverse in structure, a proportion of compounds were sufficiently similar to be grouped into chemotype clusters: Group I: JFD00244, mitoxantrone, emodin and reactive blue 2; Group II: myricetin, quercetin, morin, psoralidin, YM-26734; and Group III: BIO, GW5074, SR 27897, GSK-650394, SU 4312 (Figure 4A). The majority of top hits contained regions of high planarity, which is caused by the inclusion of aromatic rings, and in several cases, the aromatic rings were part of an extended system of conjugation (e.g. in the case of aurintricarboxylic acid - ATA, mitoxantrone and BIO). Planar or pseudo-planar nature of some hits is due to aromatic rings fused together by another ring (e.g. PD-407824). Group III was comprised of indole or indole-like compounds. In addition, hit compounds often had regions of high lipophilicity, for example, long hydrocarbon chains. Highly planar aromatic molecules are known nucleic acid binders through intercalation that explains the enrichment of these compounds in the hit-set. Another characteristic feature of the hit-set was the enrichment in compounds with a larger number of donors and acceptors organized in a 1,2 relationship (top 30 molecules when ranked by the 60-min NPD reading, e.g. JFD00244, mitoxantrone, ATA, 6-hydroxy-dl-DOPA). This arrangement is very reminiscent of the arrangement of donors and acceptors in the base pairs of RNA and therefore may facilitate Watson–Crick type bonding with the RNA base pairs. With regards to the cellular mechanism of action, the majority of hits were either inhibitors of various kinase families or receptor modulators; molecules known to modulate nucleic acid metabolism, e.g. a DNA topoisomerase II inhibitor (ATA) (57), a miRNA biogenesis inhibitor (SID 3712249) (58), and a G-quadruplex stabilising molecule (pyridostatin) (59), were also included.

Using the original FID assay, we performed a concentration response analysis for 12 hits representative of the above chemotype groups that displayed an NPD >100% (Figure 4B), with repurchased compounds where possible. Ten out of 12 hits showed a dose-response (Figure 4C, Supplementary Figure S3B). Next, compound specificity was examined using a structurally highly similar RNA, MALAT1 TH, as a substrate. Displacement from NEAT1_2 TH was found to be significantly higher for 5 out of 12 hits, and none of the compounds tested displayed higher displacement with MALAT1 TH (Supplementary Figure S4). The structure of hits was confirmed by NMR (data not shown).

Thus a screen using the NEAT1_2 TH FID assay was able to identify structural compound classes that are predicted to act as RNA binders in a diverse library of approved drugs.

### Development of a biophysical assay for validation of NEAT1_2 TH binders

FID assay provides an indirect measure of compound binding, based on a competition with the pre-bound ligand. In order to validate putative NEAT1_2 TH binders, such as those identified in our LOPAC^®1280^ screen, the use of a secondary assay that measures direct interaction is required. For such an assay we chose the recently reported waveRAPID^®^ (Repeated Analyte Pulses of Increasing Duration) approach to Grating-Coupled Interferometry (GCI) analysis (38). In contrast to the traditional kinetics, waveRAPID involves the analyte injection at a single concentration in short pulses of increasing duration followed by the longer final dissociation phase and allows efficient determination of kinetics from a single well of a 96-well plate. Recently, we have successfully utilized this approach for the analysis of protein-peptide interactions (60).

**Figure 5.**
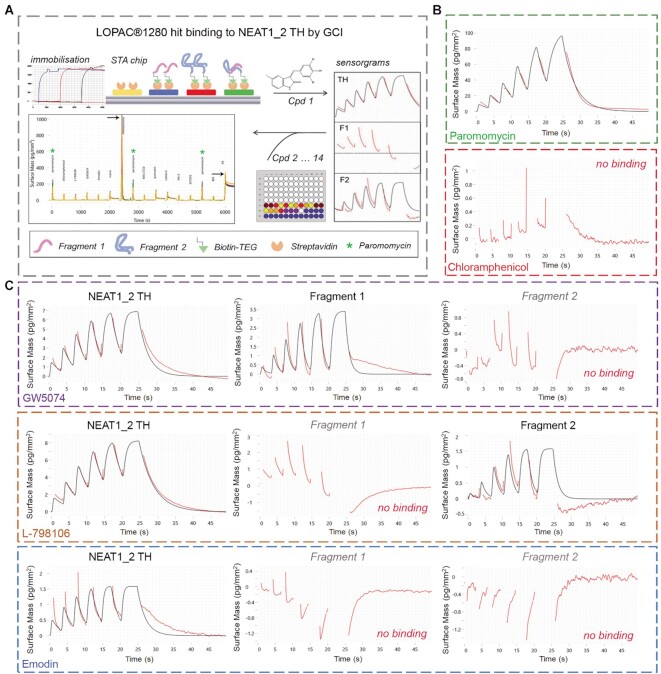
Analysis of NEAT1_2 TH binding kinetics for LOPAC^®1280^ hits using grating-coupled interferometry (waveRAPID method). (**A**) Workflow for the NEAT1_2 TH binding kinetics analysis. 5′ biotin-TEG labelled NEAT1_2 TH and individual RNA oligonucleotides were immobilized on a streptavidin-coated sensor (one channel left blank) and binding of 12 LOPAC^®1280^ hits and 2 FID assay control compounds (paromomycin and chloramphenicol) was analysed at a single concentration of 10 μM with the waveRAPID method. Compounds were injected for 25 s total injection duration followed by 300 s dissociation. Paromomycin (green asterisks) was injected three times during the run to ensure that all compounds were tested under comparable conditions. Note that mitoxantrone produced an abnormally high peak and aurintricarboxylic acid (ATA, tested last) failed to dissociate (peaks for both compounds indicated with arrows). (**B**) Sensorgrams and fits for NEAT1_2 TH binding by FID assay controls paromomycin and chloramphenicol. The double-referenced response data (red) are fitted with a binding model (black lines). Raw data were analysed and fitting performed using ‘intermediate binders’ settings and one-to-one binding model. Data from a representative experiment are shown. (**C**) Sensorgrams and fits for selected LOPAC^®1280^ hits displaying different binding affinity and specificity for NEAT1_2 TH and individual RNA oligonucleotides. Note that GW5074 binds to NEAT1_2 and fragment 1 but not fragment 2; L-789106 binds to NEAT1_2 TH and fragment 2 but not fragment 1; and emodin binds to NEAT1_2 TH but not the individual fragments. Data from a representative experiment are shown.

A biotinylated NEAT1_2 TH complex suitable for immobilisation on the streptavidin sensor surface was prepared using 5′ biotin-TEG modified fragments 1 and 2. Inclusion of the triethyleneglycol (TEG) extended spacer arm increases the oligonucleotide-biotin distance to 15 atoms and helps avoid steric hindrance issues in surface coupling assays. Importantly, 5′ ends of both fragments are located at the periphery of the TH complex (the base and the apical loop) and thus their modification was predicted to have a minimal effect on the complex. Biotinylated fragments 1 and 2 were tested alongside NEAT1_2 TH complex, to examine the potential binding specificity. Compounds were analysed at a single concentration of 10 μM, with the positive FID assay control paromomycin injected three times during the experiment as a reference, to ensure equal conditions for all compounds (Figure 5A).

Using this setup, binding of NEAT1_2 TH was confirmed for 7 out of 12 LOPAC^®1280^ hits as well as paromomycin; the negative FID assay control chloramphenicol did not bind (Figure 5B, C; Supplementary Table S2). ATA was found to poorly dissociate from RNA, whereas mitoxantrone resulted in a grossly abnormal reading (Figure 5A) therefore both molecules were excluded from replicate experiments. Although the majority of compounds tested, including paromomycin, showed binding not only to NEAT1_2 TH but also to fragment 1 and/or fragment 2, estimated *K*_d_ values for NEAT1_2 TH were typically lower than for individual fragments, indicative of the compound's higher affinity for the TH complex (Supplementary Table S2). Excitingly, we identified one compound binding NEAT1_2 TH but not the individual fragments (emodin), as well as two compounds binding NEAT1_2 TH and only one of the two fragments (L-798106 and GW5074), with the estimated *K_d_* values for the TH complex in the low micromolar range for all three compounds (2.28, 2.37 and 3.4 μM, respectively, Figure 5C, Supplementary Table S2). Interestingly, rottlerin showed binding to the individual RNA fragments but not to the TH complex (Supplementary Table S2). We also noted that most of the compounds lacking specificity over MALAT1 TH in FID assay (Supplementary Figure S4) showed no binding or abnormal binding by GCI (mitoxantrone, ATA) or did not show dose-response in FID assay (XCT790).

To conclude, several LOPAC^®1280^ hits identified using NEAT1_2 TH FID assay were confirmed in a direct binding assay.

### Cellular assay development for NEAT1_2/paraspeckles

**Figure 6.**
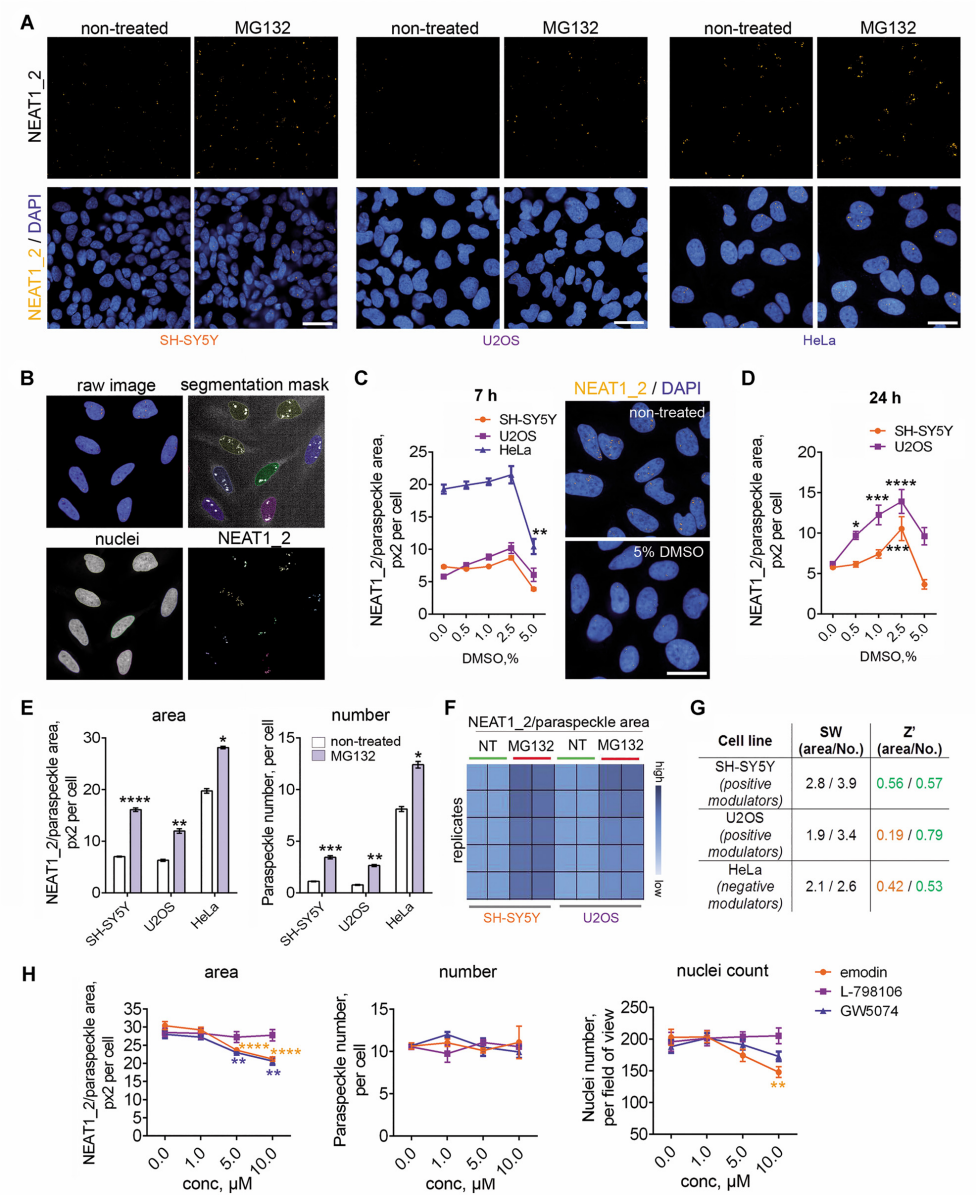
Development of a cellular assay (‘ParaQuant’) for NEAT1_2/paraspeckles and its use for validation of selected LOPAC^®1280^ hits. (**A**) Paraspeckle visualisation in three stable cell lines in a 96-well format on a high-content imaging system using a commercial RNA-FISH probe. To induce NEAT1_2/paraspeckle accumulation, cells were treated with a known paraspeckle enhancer MG132 for 4 h. NEAT1_2/paraspeckles were visualized by RNA-FISH with a NEAT1_2 Stellaris^®^ probe. Images were captured using Operetta CLS confocal system/Harmony software (40x objective lens), and maximum intensity projection images from three planes are shown. Scale bar, 20 μm. (**B**) Representative segmentation masks for paraspeckles and nuclei used for automated paraspeckle quantification. Raw image is a merged NEAT1_2 (Cy3) and DAPI image. Images are for HeLa cells. (**C**) Effect of short-term DMSO treatment on paraspeckles in three stable cell lines. Cells were exposed to increasing DMSO concentrations for 7 h; data from a representative experiment were plotted. *N* = 4 for all time-points and cell lines; **P< 0.01 (one-way ANOVA). Representative images of HeLa cells treated with 5.0% DMSO for 7 h are also shown. Scale bar, 20 μm. (**D**) Effect of prolonged DMSO treatment on paraspeckles in stable cell lines with low basal paraspeckles. Cells were exposed to increasing DMSO concentrations for 24 h; data from a representative experiment were plotted. *N* = 4 for all time-points; *P< 0.05, ***P< 0.001, ****P< 0.0001 (one-way ANOVA). (**E**, **F**) Quantification of the MG132 effect on NEAT1_2/paraspeckles in the three cell lines used for assay development. Cells were treated with MG132 for 4 h. *N* = 8, 6 and 10 for SH-SY5Y, U2OS and HeLa cells, respectively; *P< 0.05, **P< 0.01; ***P< 0.001, ****P< 0.0001 (Mann–Whitney *U* test). Note low variability between the replicate wells and heatmap suitability for the initial analysis of the compound effect (F). (**G**) *Z*’ and signal window (SW) for the three cell lines used in the assay development for positive or negative NEAT1_2/paraspeckle modulation, with MG132 and DMSO used as positive assay controls, respectively. (**H**) Effect of the selected LOPAC^®1280^ hits on NEAT1_2/paraspeckles in HeLa cells. Cells were treated with compounds at the indicated concentration for 24 h and analysed using ParaQuant. *N* = 4, **P< 0.01, ****P< 0.0001 (one-way ANOVA with Dunnett's post-hoc test). Nuclei count as a measure of toxicity is also shown.

Both *in vitro* assays described above inform on the compound binding but not on the direction of target modulation in cells, therefore the cellular activity of compounds was to be established. Scalable cellular assays suitable for rapid and cost-efficient analysis of NEAT1_2/paraspeckle levels in cultured cells are currently lacking. High local concentration of NEAT1_2 within paraspeckles provides high signal intensity in RNA *in situ* hybridisation (RNA-FISH), and excellent commercial FISH probes for NEAT1_2 are available (40,61). Taking advantage of these facts, we proceeded to develop an assay based on RNA-FISH in fixed cells. Three stable cell lines, HeLa, U2OS, and SH-SY5Y, were used, that are characterized by different levels of NEAT1_2/paraspeckles (40,62). NEAT1_2/paraspeckles could be successfully visualized with a Stellaris® NEAT1 probe, that detects NEAT1_2/paraspeckles but not the shorter NEAT1 isoform, in a 96-well format on the high-content confocal imaging system (Operetta CLS, Figure 6A, ‘non-treated’ panels; Supplementary Figure S5A). In concert with the previous studies, unstimulated SH-SY5Y and HeLa lines were displayed low and high NEAT1_2 RNA-FISH signal, respectively. U2OS cultures were highly variable, with ∼15% cells displaying high NEAT1_2 signal/multiple paraspeckles, with no or few paraspeckles detected in the rest of the cell population (Figure 6A; Supplementary Figure S5A).

Paraspeckles could be accurately detected using the ‘Spot analysis’ tool, as judged from the segmentation mask (Figure 6B). For paraspeckle quantification, two readouts were selected, ‘NEAT1_2/paraspeckle area’ and ‘paraspeckle number’. Our previous studies suggest that ‘NEAT1_2/paraspeckle area’ metric accurately reflects the total NEAT1_2 level in most cellular contexts (26,40). Certain cellular states are characterized by the accumulation of multiple individual paraspeckles, e.g. when TDP-43 protein is depleted (26),—in such cases, the ‘paraspeckle number’ metric is also informative. However, individual paraspeckles often cluster together and hence are counted as a single paraspeckle, leading to an underestimated ‘paraspeckle number’ value, therefore this metric cannot be used as the sole assay readout. Overall, with the combined use of the two readouts, the majority of paraspeckle phenotypes will be captured.

Using the above quantification approach, we first determined the DMSO sensitivity of NEAT1_2/paraspeckles, which was not previously reported. Short-term (7-h) exposure to 0.5–2.5% DMSO led to only slight, non-significant increase in NEAT1_2/paraspeckle area in all three cell lines (Figure 6C). However, a dramatic drop in NEAT1_2/paraspeckle area was recorded at 5.0% DMSO in HeLa cells, despite the lack of obvious changes in the cellular/nuclear morphology (Figure 6C). Strikingly, pre-treatment with 5.0% DMSO also almost completely abrogated MG132-induced NEAT1_2 accumulation (Supplementary Figure S5B). Prolonged (24-h) treatment with 0.5–2.5% DMSO led to a concentration-dependent increase of NEAT1_2/paraspeckle area in cell lines with ‘low’ basal paraspeckles, SH-SY5Y and U2OS, however at 5.0% DMSO, no difference from the baseline was observed (Figure 6D). MG132 is a reliable NEAT1_2/paraspeckle inducer with a rapid effect and minimal toxicity with short (∼4 h) incubations (6) therefore we tested its utility as a positive assay control. As expected, MG132 robustly induced NEAT1_2/paraspeckles in SH-SY5Y and U2OS cells however this effect was less obvious in HeLa cells (fold change < 2; Figure 6A,E,F). The results with DMSO and MG132 treatments suggested that cell lines with scarce paraspeckles such as SH-SY5Y and U2OS would be most suitable for the analysis of positive paraspeckle modulators, whereas cell lines with prominent/abundant paraspeckles such as HeLa would be preferred for the identification of paraspeckle inhibitors.

*Z*’ and signal window (SW) statistics were calculated for SH-SY5Y and US2OS cells using MG132 as the positive assay control separately for the NEAT1_2/paraspeckle area and paraspeckle number metrics (Figure 6G). These assay statistics were also calculated for HeLa cells by using 5.0% DMSO that almost eliminates paraspeckles, as the positive control (Figure 6G). *Z*’ values were typically above or close to 0.5 indicating adequate assay performance, as per the Broad Institute's guidelines for image-based high-content screening assays (63).

Subsequent assay optimisation activities were aimed at increasing its cost-efficiency and reducing assay setup time. It was established that quantification-compatible images of NEAT1_2/paraspeckles can be obtained with at a final probe concentration of as little as 10 nM, where increased the density of cells or use of cells with more prominent paraspeckles did not require increased probe concentrations (data not shown). Reducing probe incubation times to 6 h and reducing post-hybridisation washes (single 30 min wash in phosphate buffered saline) also did not affect the image quality and hence their quantification. Imaging did not require mounting media or mineral oil and could be successfully performed in phosphate buffered saline. Overall, the total assay time, from cell fixation to imaging data and with 2–4 plates processed simultaneously, was ∼8 h, with the researcher time at bench being less than 60 min. Although the initial assay development was performed using Operetta CLS, the assay is compatible with the majority of other automated/high-content imaging systems, e.g. IN Cell Analyzer and ImageXpress Micro (data not shown). Overall, this assay that we dubbed ‘ParaQuant’ is suitable for rapid screening for positive and negative modulators of paraspeckles in various adherent cell lines in at least 96-well format.

We next used this assay to test potential cellular activity of the LOPAC^®1280^ hits. NEAT1_2/paraspeckles are highly sensitive to cellular stress with a variety of treatments increasing their levels (61,64), therefore the use of compounds with confirmed toxicity may lead to non-specific accumulation of paraspeckles. LOPAC^®1280^ hits were first tested for toxicity in SH-SY5Y cells using a resazurin-based viability assay. Upon a 24-h treatment, the majority of hits were not toxic at concentrations of up to 10 μM, with the exception of mitoxantrone which caused reduction in cellular viability at concentrations ≥ 200 nM (Supplementary Figure S6A). A 24-h mitoxantrone treatment at concentrations of ≥50 nM led NEAT1_2/paraspeckle accumulation in SH-SY5Y cells, as evidenced by a significant increase in both paraspeckle metrics (Supplementary Figure S6B–D). This was accompanied by a significant decrease of the nuclei count (Supplementary Figure S6E), in agreement with the resazurin-based assay data, suggesting that the compound's effect on paraspeckles is largely due to a non-specific toxicity. This experiment also demonstrated that the nuclei count can be successfully used as a toxicity readout within the ParaQuant. Rottlerin, that similar to mitoxantrone did not show specific TH binding by GCI, was also found to promote paraspeckle accumulation while reducing nuclei count (data not shown).

The three compounds with NEAT1_2 TH binding verified by GCI were tested by ParaQuant (24-h exposure): emodin (TH binding only); GW5074 (TH and fragment 2); L-798106 (TH and fragment 1). Whilst no effect was observed in SH-SY5Y cells (data not shown), in HeLa cells, emodin and GW5074 at higher concentrations were able to decrease NEAT1_2/paraspeckle area by 30.3%/27.3% and 26.3%/24.1% (5 μM/10 μM), respectively, without affecting paraspeckle numbers, and L-798106 did not have a significant effect on paraspeckles (Figure 6H). Emodin was however moderately toxic, as indicated by a decreased nuclei count (Figure 6H). Thus two out of three compounds identified as NEAT1_2 TH binders using a combination of *in vitro* assays were confirmed as having a cellular activity.

### Validation of ParaQuant as a primary screening assay using a kinase inhibitor library

**Figure 7.**
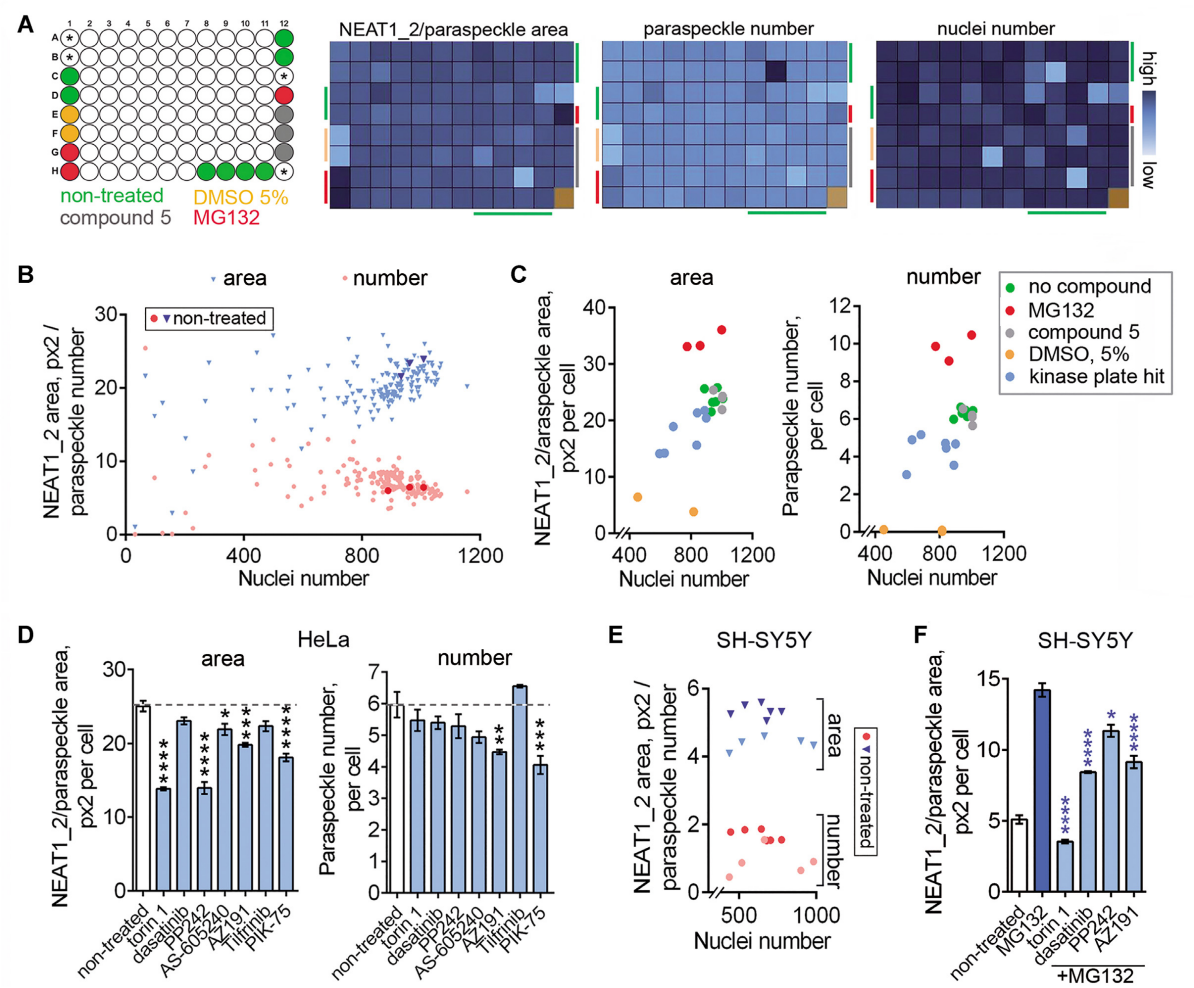
Validation of ParaQuant as a primary screening assay (HeLa cells) and identification of kinase inhibitors – negative paraspeckle modulators. (**A**) Plate layout and examples of heatmaps for a Cayman Chemical Kinase Library screening plate. Three assay controls were included, MG132 (6.5 μM for 4 h, paraspeckle enhancer), DMSO (5.0% for 7 h, paraspeckle inhibitor) and compound 5 (5 μM for 24 h, no effect on paraspeckles). Unused wells are indicated with asterisks. White wells were treated with the library compounds. Location of controls on the heatmaps is indicated with a bar of respective colour. Results for the library plate 2 in HeLa cells are shown. (**B**) Correlation plot for the paraspeckle readouts (area and number) combined, *vs*. nuclei count for the Cayman Chemical Kinase Library screen. Nuclei count per 10 fields of view was plotted. (**C**) Correlation plots for paraspeckle readouts (area and number) *vs*. nuclei number for the kinase library hits taken into retest as compared to assay controls (‘zoom-in’ from B). (**D**) Retest results for the kinase inhibitor library hits. Compounds were tested with the original assay conditions (5 μM for 24 h). *N* = 3–4; *P< 0.05, **P< 0.01, ***P< 0.001, ****P< 0.0001 (one-way ANOVA with Dunnett's post-hoc test). (**E**) Negative modulators of paraspeckles identified in HeLa cells are also active in SH-SY5Y cells. Compound data from the original kinase inhibitor library screen (Supplementary Table S3) were plotted. Nuclei count per 10 fields of view was plotted. (**F**) Negative modulators of paraspeckles identified in HeLa cells are able to prevent or attenuate MG132 induced paraspeckle accumulation in SH-SY5Y cells. Kinase inhibitor library hits were tested in duplicates. Cells were treated with the inhibitor (5 μM) for 20 h and subsequently subjected to MG132 (6.5 μM) for an additional 4 h, in the presence of the compound. Compounds were tested in duplicates; *P< 0.05, ****P< 0.0001 (one-way ANOVA with Dunnett's post-hoc test, as compared to MG132).

To ascertain the suitability of ParaQuant for applications with a higher throughput, we used it to screen a small library. Given the sensitivity of NEAT1_2/paraspeckle levels to cellular stress and a central role of protein kinases in stress signaling, a kinase inhibitor library was selected. Cayman Chemical Kinase Screening Library that includes ∼160 selective and non-selective kinase small molecule inhibitors for 70 distinct kinases and kinase families was screened in HeLa and SH-SY5Y cells at 5 μM, with a 24-h treatment. Since we observed only a small variation between the replicates during assay development (Figure 6E, F), the library was screened in single replicates. ParaQuant images for all wells were inspected for any artefacts affecting paraspeckle quantification. All compounds passed this manual filter except bisindolylmaleimide IX that produced a bright cytoplasmic Cy3 signal and was excluded from further analysis; this compound is red in colour and its intracellular accumulation likely produced this artefact. *Z*’ for the two kinase library plates (both NEAT1_2 area and paraspeckle number metrics) ranged between 0.58 and 0.75. We first focused on the results in HeLa cells expected to yield negative modulators of paraspeckles. Heatmaps for paraspeckle readouts and nuclei count were found suitable for rapid initial analysis of the screening outcomes, as demonstrated in Figure 7A. A subset of compounds (12.3%) showed significant toxicity as evidenced by >40% reduction of the nuclei count (Figure 7A, B; Supplementary Table S3). We selected seven compounds that caused >20% decrease in paraspeckle number, paraspeckle area, or both, and low-to-moderate (<35% of control) reduction in the nuclei count (Figure 7C, ‘zoom-in’ from Figure 7B); these compounds were retested in triplicates. In retest, five out of seven hits were confirmed to significantly reduce NEAT1_2/paraspeckle area and/or number (Figure 7D). All compounds displayed a varying extent of cellular toxicity in the retest (13–48% reduction in the nuclei count), which was not surprising given the high concentration and treatment duration used. Encouragingly, in SH-SY5Y cells, four out of these five compounds also caused a reduction in both NEAT1_2/paraspeckle area and number with limited or no toxicity (original screen, Figure 7E). Four out of seven primary hits were also able to prevent or significantly attenuate MG132-induced paraspeckle accumulation in SH-SY5Y cells (Figure 7F), with the remaining compounds being highly toxic upon co-treatment with MG132.

In SH-SY5Y cells, we focused on paraspeckle-inducing compounds. As expected, the majority of compounds that induced paraspeckles were toxic, as indicated by a significantly reduced (<60% of control) nuclei count (Supplementary Figure S7A). We selected 5 hits that displayed >1.5-fold increase in paraspeckle numbers and low toxicity (nuclei count >85% of control) for retest; a lower concentration (500 nM) was also included (Supplementary Figure S7B). Four out of five compounds provided at least >2-fold increase in paraspeckle number at 5 μM however none of these retained the activity at 500 nM (Supplementary Figure S7C). Only one compound, the MLK3 inhibitor URMC-099, was not toxic at the active (5 μM) concentration (Supplementary Figure S7C).

Finally, we miniaturized the ParaQuant assay to a 384-well format and validated this format by retesting the kinase inhibitor hits obtained in HeLa and SH-SY5Y cells. Average *Z*’ values for NEAT1_2/paraspeckle area for HeLa and SH-SY5Y cells in this experiment were 0.46 and 0.53, respectively, and hit activity results (NEAT1_2/paraspeckle modulation) were in agreement with those obtained in the 96-well format (Supplementary Figure S8).

### Identification of dual PI3K/mTOR inhibitors as negative modulators of NEAT1_2/paraspeckles

**Figure 8.**
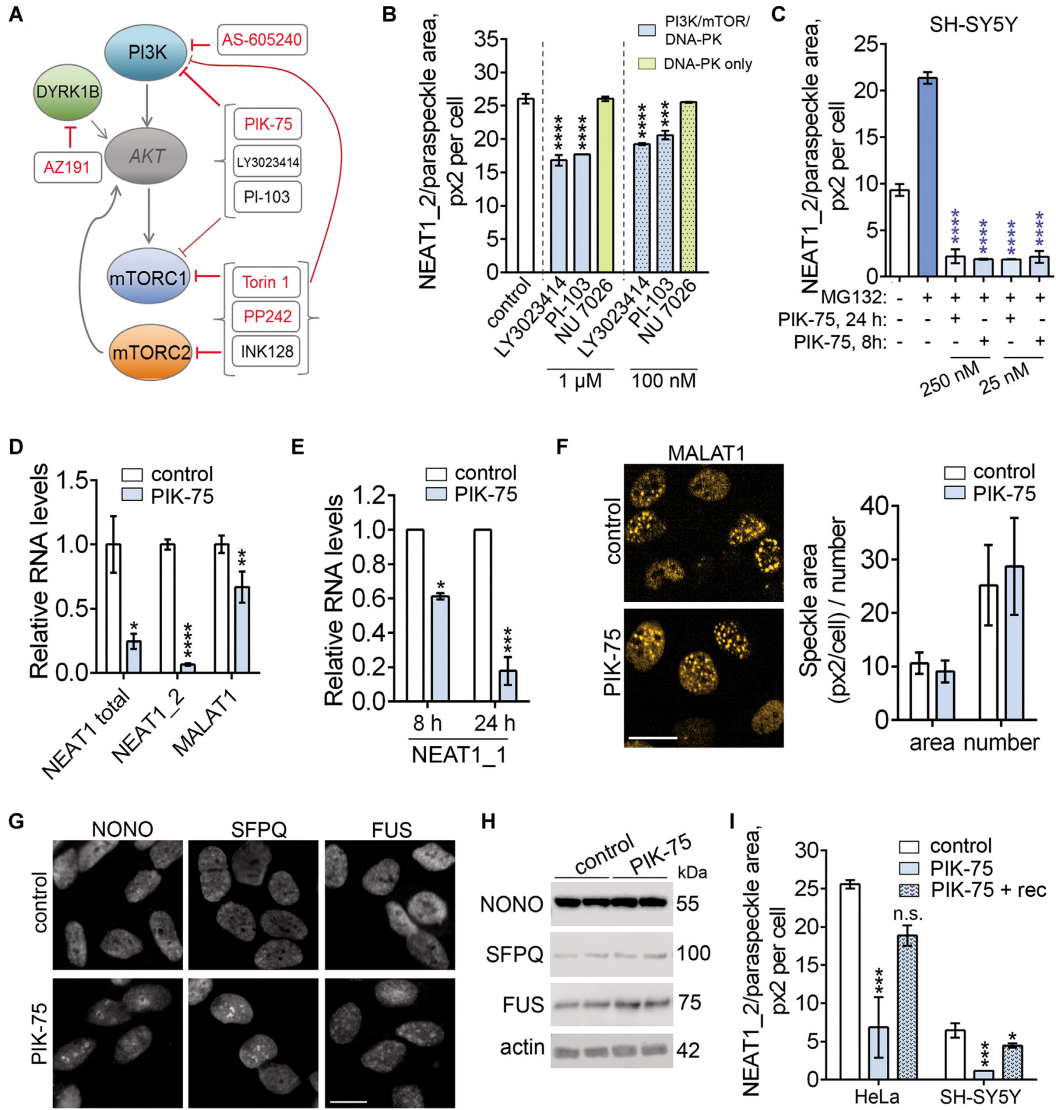
Characterisation of negative modulators of NEAT1_2/paraspeckles. (**A**) Negative modulators of paraspeckles identified in the kinase inhibitor screen act within the PI3K/mTOR signalling pathway. Hits from the screen in HeLa cells are shown in red and additional dual PI3K/mTOR inhibitors tested are given in black. (**B**) PI3K/mTOR/DNA-PK inhibitors but not a specific DNA-PK inhibitor downregulate NEAT1_2/paraspeckles in HeLa cells. Compounds were tested in duplicates at the indicated concentration using the ParaQuant setup. Cells were treated with the inhibitor for 24 h. ***P< 0.001, ****P< 0.0001 (one-way ANOVA with Dunnett's post-hoc test). (**C**) PIK-75 abolishes MG132 induced paraspeckle accumulation in SH-SY5Y cells. Cells were treated with PIK-75 for 20 h or 4 h at the indicated concentration and subsequently subjected to MG132 (6.5 μM) for an additional 4 h. ****P< 0.0001 (one-way ANOVA with Dunnett's post-hoc test, as compared to MG132). (**D**) PIK-75 downregulates total NEAT1 and NEAT1_2 but has limited effect on MALAT1 lncRNA as measured by qRT-PCR. SH-SY5Y cells were treated with 250 nM PIK-75 for 24 h. *N* = 4 or 6. *P< 0.05, **P< 0.01, ****P< 0.0001. (**E**) PIK-75 downregulates NEAT1_1 as measured by qRT-PCR. NEAT1_2 knockout SH-SY5Y cells (see Supplementary Figure S11) were treated with 250 nM PIK-75 for 8 or 24 h. *N* = 4. *P< 0.05, ***P< 0.001. (**F**) PIK-75 has limited effect on MALAT1/speckles. HeLa cells were treated with 25 nM PIK-75 for 24 h, and MALAT1 was detected by RNA-FISH and quantified using Spot analysis on Harmony software (*N* = 4). Representative images are also shown. Scale bar, 20 μm. (**G**) PIK-75 causes redistribution of essential paraspeckle proteins to the perinucleolar regions (SFPQ and NONO) or speckle-like territories (FUS). SH-SY5Y cells were treated with 250 nM PIK-75 for 24 h. Scale bar, 10 μm. (**H**) PIK-75 does not affect the levels of core paraspeckle proteins as determined by western blot. SH-SY5Y cells were treated with 250 nM PIK-75 for 24 h. (**I**) PIK-75 effect on NEAT1_2/paraspeckles is reversible. HeLa and SH-SY5Y cells were treated with PIK-75 at 25 or 250 nM respectively, for 24 h, followed by recovery in compound-free media for 16 h. *N* = 3–4, *P< 0.05, ***P< 0.001 (one-way ANOVA with Dunnett's post-hoc test).

Since the field is currently critically short of paraspeckle inhibitors, we further focused on the hits from the HeLa cell screen. We noticed that two out of five compounds confirmed in retest in HeLa cells, Torin 1 and PP242, are potent and selective mTORC1/2 inhibitors (‘TORkinibs’) (65). Consistently, the third TORKinib, INK128, also decreased NEAT1_2 positive area by 26.4% (but not paraspeckle number) in the original screen in HeLa cells (Supplementary Table S3). The remaining three compounds, PIK-75, AS-605240 and AZ191, are the inhibitors of PI3K p110α, PI3K p110γ and DYRK1B, respectively. PIK-75 also inhibits mTORC1/2 at ≥1 μM (66), whereas DYRK1B was reported to activate mTOR (67). TORKinibs also inhibit PI3K. Thus negative modulators of paraspeckles identified in this screen act at the level of the PI3K/Akt/mTOR pathway (Figure 8A). The activity of TORKinibs and PIK-75 was verified using compounds from an independent batch/provider, including after a short treatment (8 h) (Supplementary Figure S9A). However most of the hits have broader activity, at least in higher concentrations, – in particular, PIK-75, Torin 1 and PP242 inhibit DNA-PK (summarized in Supplementary Figure S9B). Re-examination of the screen results both in HeLa and SH-SY5Y cells revealed that selective PI3K inhibitors TGX-221 (p110β) and CAY10505 (p110γ) had no effect on paraspeckles, whereas dual PI3K/mTOR inhibitors GSK1059615 and CAY10626 as well as a PI3K/mTOR/DNA-PK inhibitor PI-103 all decreased either NEAT1_2 area or paraspeckle number at least in one cell line. At the same time, a dual PI3K/DNA-PK inhibitor wortmannin and a selective DNA-PK inhibitor NU 7026 had no effect on paraspeckles or upregulated them, and none of the Akt inhibitors in the library affected paraspeckles (Supplementary Table S3; Supplementary Figure S9B; Figure 8B). An additional dual PI3K/mTOR inhibitor LY3023414 (samotolisib) tested also significantly downregulated paraspeckles (Figure 8B). Thus a phenotypic screen using ParaQuant assay identified dual PI3K/mTOR inhibitors as potent negative modulators of paraspeckles.

To corroborate this finding, we performed depletion of core components of the four complexes under investigation – raptor (*RPTOR*; mTORC1), rictor (*RICTOR*; mTORC2), p110α (*PI3KCA*) and DNA-PKcs (*PRKDC*) using specific siRNAs in HeLa cells (Supplementary Figure S10A). Raptor and p110α, but not rictor depletion led to modest but significant decrease in NEAT1_2/paraspeckle area (Supplementary Figure S10B). Rictor-depleted cells however had increased numbers of smaller paraspeckles (Supplementary Figure S10C), suggesting a role of mTORC2 in paraspeckle maintenance. Raptor and p110α knockdown did not have an additive effect confirming that the two factors act within the same pathway. On the contrary, DNA-PKcs knockdown led to significant upregulation of NEAT1_2/paraspeckles (Supplementary Figure S10B), potentially due to DNA damage toxicity. Although PI3K and mTOR regulate cell cycle, no differences in cell proliferation after knockdown of these factors were observed (data not shown). Thus the PI3K-mTOR axis contributes to the maintenance of basal paraspeckle levels.

We next focused on PIK-75 as the most potent paraspeckle inhibitor (Supplementary Figure S9A), aiming to gain insights into its mechanism of action and further establish it as a tool for paraspeckle research. We noted that the repurchased compound was significantly more potent than the library-supplied one, probably due to partial degradation; its activity against paraspeckles was additionally verified using a compound batch from a third provider (data not shown). Dose–response and survival analyses revealed that PIK-75 is significantly more toxic in HeLa as compared to SH-SY5Y cells, and established optimal (activity *vs*. toxicity) concentrations of 25 and 250 nM for HeLa and SH-SY5Y cells, respectively. PIK-75 at low concentrations was able to completely prevent MG132-induced paraspeckle assembly in SH-SY5Y cells (Figure 8C). Downregulation of total NEAT1 (NEAT1_1 + NEAT1_2) and NEAT1_2 in PIK-75 treated cells was verified by qRT-PCR (Figure 8D). Since ParaQuant or qRT-PCR cannot distinguish between the two NEAT1 isoforms in WT cells, we used a paraspeckle-deficient SH-SY5Y line that we recently generated (Supplementary Figure S11). NEAT1_1 was also found significantly downregulated by PIK-75 (Figure 8E). In contrast, levels of MALAT1 lncRNA transcribed from a nearby locus and often regulated in the same direction as NEAT1 (68) were affected to a much lesser extent (Figure 8D). Consistently, MALAT1-positive speckles visualized by RNA-FISH remained intact after PIK-75 treatment (Figure 8F). We next examined the effect of PIK-75 on the levels and distribution of core paraspeckle proteins SFPQ and NONO that are known to stabilize NEAT1_2 (69). Both proteins were found to relocate to perinucleolar regions; interestingly, FUS protein required for higher-order assembly of paraspeckles (70) appeared in a speckled pattern or was condensed around the nucleolus in PIK-75 treated cells (Figure 8G). Levels of these proteins remained unchanged even after prolonged PIK-75 treatment (Figure 8H). PIK-75′s effect on NEAT1_2/paraspeckle levels and paraspeckle protein distribution was reversible even after prolonged exposure (24 h/16 h recovery; Figure 8I; Supplementary Figure S10D). Thus, PIK-75 triggers redistribution of paraspeckle proteins in the nucleus, downregulation of both NEAT1 isoforms and paraspeckle loss, and this phenotype can be reversed.

### Multiplexing with ParaQuant for the analysis of additional cellular phenotypes

**Figure 9.**
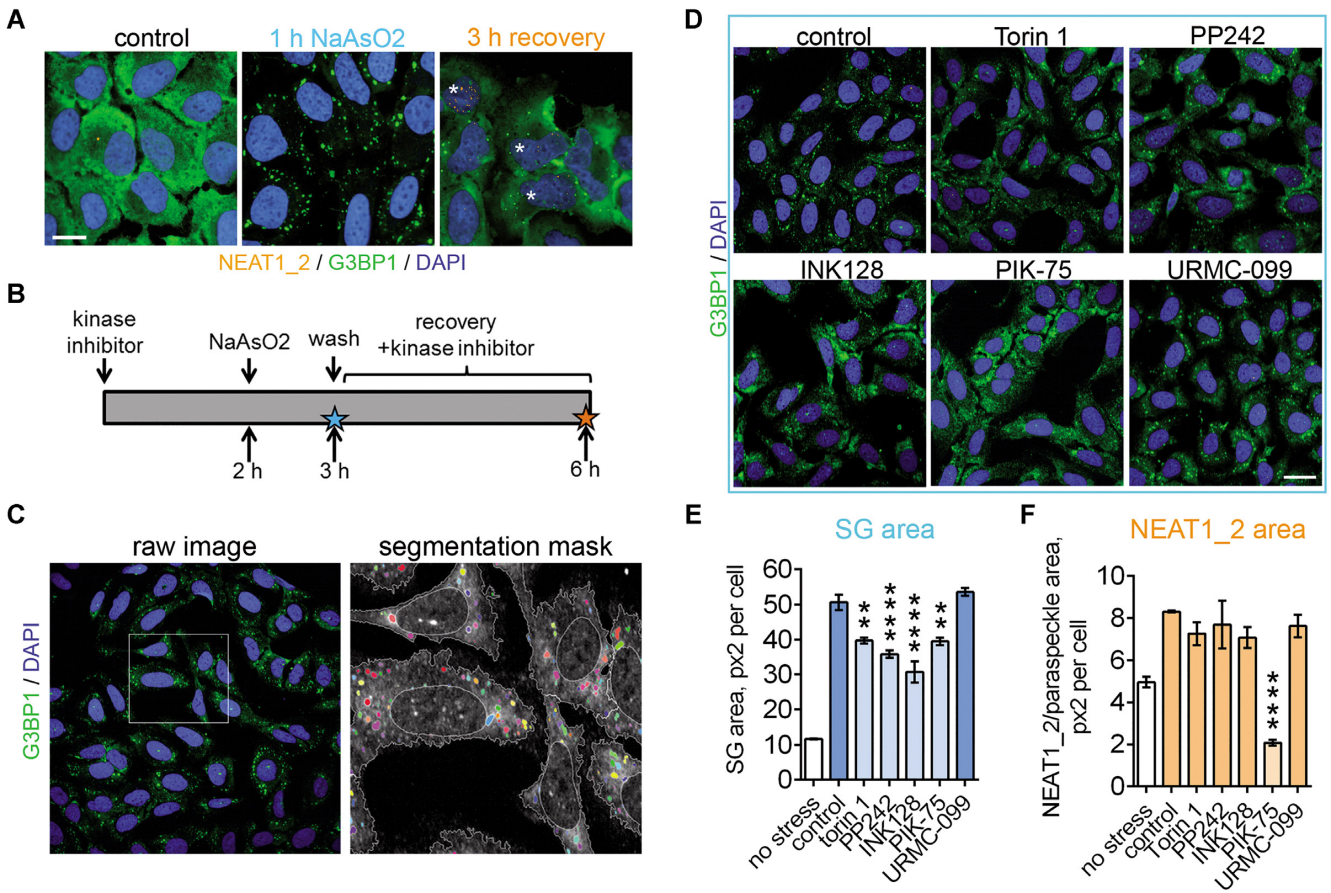
Multiplexing and analysis of additional cellular phenotypes with ParaQuant assay: stress granules. (**A**) Simultaneous detection of paraspeckles and stress granules (SGs) in U2OS cells. Representative images of paraspeckles and SGs in cells subjected to NaAsO_2_ and analysed at the early and late time-points during stress (1 h stress and 3 h recovery in stressor-free media, respectively). Paraspeckle visualisation was performed as described for ParaQuant assay and SGs were visualized with CoraLite®488-conjugated anti-G3BP1 antibody. Nuclei with prominent paraspeckles are indicated with asterisks. Scale bar, 10 μm. (**B**) Timeline for the kinase inhibitor experiment. Stars indicate time-points for SG and NEAT1_2/paraspeckle analysis. (**C**) Representative segmentation mask for SGs used for automated quantification of SG area. Raw image is a merged G3BP1 and DAPI image. (**D**) Representative images of SG assembly in U2OS cells pre-treated with the indicated kinase inhibitors. Cells were pre-treated with the inhibitor for 2 h (5 μM) and subjected to NaAsO_2_ for 1 h. Scale bar, 20 μm. (**E**, **F**) SG area (E) and NEAT1_2/paraspeckle area (F) analysed at the early (1 h NaAsO2) and late (3 h recovery) time-points, respectively. **P< 0.01, ****P< 0.0001 (one-way ANOVA with Dunnett's post-hoc test).

Next we tested whether the ParaQuant assay can be used with multiplexing and combined with the analysis of other cellular phenotypes, including in more complex models. We previously reported that under certain stress conditions, NEAT1_2/paraspeckles are regulated by cytoplasmic RNP granules stress granules (SGs). The two types of granules display different temporal dynamics where SGs assemble early during stress and positively regulate paraspeckle formation late in stress or during the recovery phase, as was established using NaAsO_2_ (sodium arsenite) as a model stressor (61). We investigated whether ParaQuant can be used for simultaneous SG and paraspeckle analysis. U2OS cells were selected as they are commonly used for SG analysis. Inclusion of a 30-min incubation step with a fluorescently-tagged antibody for a standard SG marker G3BP1 allowed to efficiently label SGs without affecting the NEAT1_2/paraspeckle signal (Figure 9A). PIK-75 was previously shown to inhibit SG assembly (71). This compound, the three TORKinibs and URMC-099 were tested under the conditions of SG-inducing stress. Cells were pre-treated with the inhibitor for 2 h, stressed with NaAsO_2_ for 1 h (peak of SG accumulation), washed and left to recover in the presence of inhibitor for 3 h (peak of NEAT1_2/paraspeckle accumulation). SG and NEAT1_2/paraspeckle area were quantified after 1 h of NaAsO_2_ treatment and 3 h into the recovery (Figure 9B, C). Short, 2-h treatment with PIK-75 and TORKinibs, but not URMC-066, negatively affected SG assembly as evidenced by diminished SG area (Figure 9D, E). Under these conditions, PIK-75 dramatically reduced NEAT1_2/paraspeckle area at the 3-h recovery time-point (Figure 9F). Other compounds also had a mild negative effect on paraspeckles which was not significant. Therefore, PIK-75 is a potent inhibitor of NEAT1_2/paraspeckles under SG-inducing stress where its activity may be at least partially mediated by its modulatory effect on SGs.

Finally, we assessed the suitability of this assay for the studies of the NEAT1_2/paraspeckle response to stress in human motor neurons differentiated from stem cells. To take advantage of the multiplexing option, we included an additional marker (beta-III tubulin) to monitor the integrity of the neuronal network. NEAT1_2/paraspeckles are virtually absent in postmitotic neurons (26), but could be induced by NaAsO_2_ treatment with a 24-h recovery (72). By using ParaQuant in a 96-well format and neurite detection by anti-beta-III tubulin staining, we were able to simultaneously quantify the NaAsO_2_ induced NEAT1_2/paraspeckle accumulation and disruption of the neurite network (Supplementary Figure S12).

Therefore, ParaQuant is compatible with simultaneous capture and analysis of additional cellular phenotypes in various cellular contexts.

## DISCUSSION

NEAT1 is a molecule of exceptional therapeutic significance, especially for oncology indications, however currently no chemical ligands are known to specifically bind and modulate this lncRNA. Furthermore, our knowledge of compound classes that can modulate NEAT1_2/paraspeckle levels indirectly is also very limited, impeding efficient research into the signalling cascades controlling paraspeckle abundance and function. NEAT1 isoforms are transcribed from the same promoter and have an overlapping segment, yet they have different and in some contexts, opposite cellular functions (4). Therefore the ability to selectively alter NEAT1_2 levels posttranscriptionally would open multiple avenues for manipulation of the isoform-specific pathological functions. Our current study provides novel methodological resources, in the form of assays and small molecules, for NEAT1_2/paraspeckle research and drug discovery.

### *In vitro* assays

We used a structured motif of NEAT1_2, the 3′-terminal stability element folded into a triple-helical conformation, as a substrate for *in vitro* assay development. Such complex elements in RNA can create binding pockets and hold a great promise for RNA-focused small molecule drug discovery. Identification of such motifs is currently gaining pace due to the development of relevant databases, e.g. the Inforna (73). We demonstrate that an in-solution assay based on fluorescent dye displacement from RNA is a simple and cost-efficient assay of choice for complex three-dimensional RNA motifs. We show that NEAT1_2 TH is stable when reconstituted *in vitro* and frozen; thus it can be prepared in batches to streamline the HTS process. It is important to note that the assay is sensitive to DMSO concentrations of ≥2%. This was unsurprising given the reported effect of DMSO on RNA structure and ligand binding by competing for hydrophobic interactions (74) and should be taken into account for HTS planning for RNA targets. We found that small molecules known to bind RNA hairpins – paramomycin, G418, and LMI070 (branaplam) – result in significant dye displacement in NEAT1_2 TH FID when used at high concentrations, consistent with the presence of a double-stranded hairpin segment within the NEAT1_2 TH. Interestingly, linezolid led to a small increase in fluorescence, which may be indicative of structural changes in the RNA that result in more favourable conformations for dye intercalation, e.g. RNA becoming ‘more structured’ or the proportion of structured RNA in the sample increasing. In the LOPAC^®1280^ screen however, only two compounds yielded a similar increase in fluorescence, and the rare occurrence of such compounds confirms that the assay substrate is highly structured already.

As expected, the majority of hits from the LOPAC^®1280^ pilot screen were planar, aromatic molecules that bind nucleic acids via intercalation. In addition, a subset of hits potentially form Watson-Crick type bonding with RNA bases. The characteristics of our hit-set were consistent with the fact that RNA binders, on average, have more aromatic rings and more H bond and acceptor groups, based on the comparison of RNA binders registered in the Inforna/R-BIND databases (73,75) with FDA-approved drugs (DrugBank). For several hits, direct binding to NEAT1_2 TH was confirmed in a surface binding assay. While the molecules identified do not represent suitable starting points for further optimisation, this result is encouraging and suggests that it should be possible to identify NEAT1_2 TH ligands with optimal physicochemical properties via the use of FID assay in larger chemical collections. For example, such screens may be able to find probes that interact with specific pockets within NEAT1_2 TH, similar to the small molecule that binds in a pocket-like structure (‘site 2’) within MALAT1 TH recently identified in a *in silico* docking study (50). FID assay is easily scalable and hence suitable for HTS campaigns (>50 000 compounds) on structured RNA motifs. Additional filtering steps, at the stage of library assembly or later on, could help eliminate potentially problematic compounds (76). However given limited understanding of binding modes for this relatively new target class (structured RNA), caution should be taken during the filtering steps, to ensure that promising hits are not missed. Secondary assays with reasonable throughput can be used for post-HTS filtering. For example, waveRAPID GCI, can be applied for deconvolution of HTS results. This rapid kinetics method is a fast and convenient option for screening potential binders in a 96-well format and can be used as a primary filtering assay to shortlist compounds for subsequent detailed binding characterisation, e.g. by ITC or NMR. To the best of our knowledge, our study is the first to report the use of GCI for the analysis of RNA-ligand interactions for a structured RNA target. However, SPR can also be successfully utilized for this purpose, as evidenced by a number of published studies (77,78), therefore a biophysical assay of this kind is potentially accessible for any laboratory.

An obvious limitation of these *in vitro* assays is the use of ‘naked’ RNA, as opposed to the cellular contexts where RNA molecules rarely, if ever, are found outside RNP complexes (79). Yet, given the dynamic nature of RNP particles, specific structural elements of RNA are likely easily accessible to small molecules.

### Cellular NEAT1_2/paraspeckle assay

Cellular validation is critical for RNA targets which often demonstrate poor correlation between the results of binding analysis and functional studies. We propose that the ParaQuant assay allowing direct and highly accurate quantification of NEAT1_2 levels in a 96- or 384-well format can be used as a useful alternative to the existing NEAT1_2/paraspeckle quantification approaches. Currently, qRT-PCR is the primary approach used for NEAT1_2/paraspeckle quantification. However NEAT1_2 quantification by qRT-PCR is often unreliable due to so-called ‘semi-extractability’ of the molecule. NEAT1_2 exists in a tightly packed complex with multiple RNA-binding proteins leading to its reduced recovery during extraction with guanidine isothiocyanate solutions commonly used for RNA purification (80). Even after the inclusion of mechanical shearing and heating steps, NEAT1_2 extraction rates may still be highly variable, including between the replicates from the same experiment purified in parallel (our unpublished observations). This variability can preclude capturing subtle changes in NEAT1_2/paraspeckle abundance, where small (∼10–20%) changes in their levels may still be meaningful from the point of view of their therapeutic modulation. In addition, qRT-PCR is a multi-step labour/time-consuming process with high cost and limited throughput. ParaQuant is suitable for compound screening in single replicates, whereas qRT-PCR requires 3–4 technical repeats and inclusion of a housekeeping control gene for each sample for normalisation. ParaQuant also allows simultaneous analysis of additional markers of cellular phenotypes, such as markers of subcellular structures and toxicity markers (of which nuclei count is already captured in the basic assay setup).

Critically, ParaQuant allows morphological analysis of paraspeckles. Changes in the gene expression and signaling cascades as well as disease mutations can affect paraspeckle integrity and structure without affecting NEAT1_2 levels, as we showed here for mTORC2 (rictor) depletion and previously for ALS-FUS mutations (40). A limited number of cell models with fluorescently labelled endogenous NEAT1_2/paraspeckles, via MS2-MCP or CRISPR-dCas13 systems, have been generated (81,82). However they typically suffer from non-specific labelling of other structures in the nucleus (e.g. nucleolus) and background issues. Therefore, although highly instrumental for live imaging studies, these approaches do not provide a signal that is sufficiently ‘clean’ to support the development of a robust quantitative assay. NEAT1_2/paraspeckle quantitation via analysis of foci formed by fluorescently labelled core paraspeckle proteins such as SFPQ, NONO or FUS (71) is possible however these proteins are also recruited to other nuclear bodies or form aggregates when accumulated (83). Yet perhaps the biggest limitation to the use of engineered cell lines is that they require the resource-intensive cell line generation each time the assay is used in a new cell line, whereas ParaQuant assay can be used in any adherent cell line.

ParaQuant can be used as a primary screening assay or as a secondary assay to establish the direction of compound activity in cells (inhibitor or activator), including in hit-to-lead optimisation. Two major considerations should be taken into account when designing ParaQuant experiments, namely, paraspeckle abundance in the selected cell line and vehicle/solvent concentration. Cell lines with ‘high’ basal paraspeckles such as HeLa are more suitable for the search/analysis of paraspeckle inhibitors, whereas cells with ‘low’ basal paraspeckles, e.g. SH-SY5Y, will provide a better signal window for the identification of positive paraspeckle modulators. We found that final DMSO concentrations exceeding 0.5% should be avoided in assaying NEAT1_2/paraspeckle changes as they will typically upregulate NEAT1_2. Unexpectedly, we also found that 5.0% DMSO dramatically decreases NEAT1_2/paraspeckles and even prevents their induction by MG132. Although DMSO is highly reactive with RNA (80) and therefore this effect is likely non-specific, DMSO at this concentration can be utilized as a control treatment in screens and other experiments.

Using a phenotypic screen, we established that the PI3K/mTOR kinase cascade maintains the basal levels of paraspeckles, and its activity is also important for stress-induced NEAT1_2/paraspeckle upregulation. Previously, we showed that NEAT1_2/paraspeckles form late during stress and are positively regulated by cytoplasmic SGs (61). mTOR activity is modulated by SGs (84), and PI3K was recently reported to promote SG assembly (85). The interplay between PIK3/mTOR signalling, NEAT1_2/paraspeckles and SGs under various stress conditions warrants further detailed research.

The use of ParaQuant assay with a kinase inhibitor library led us to identify a number of potent paraspeckle inhibitors, first of all, PIK-75, and its mechanism of action was characterized. PIK-75 causes redistribution of essential paraspeckle proteins in the nucleus and hence loss of their function in paraspeckle maintenance. Both NEAT1 isoforms are downregulated whereas MALAT1 and speckles were only minimally affected in the presence of PIK-75. Paraspeckle depletion by PIK-75 is reversible. Given the lack of chemical inhibitors of paraspeckles, these compounds should be instrumental in probing the biological and pathological functions of paraspeckles in various systems. Likewise, novel compounds that upregulate NEAT1_2/paraspeckles identified here (mitoxantrone, rottlerin) can be utilized in paraspeckle research, assay development and screens.

In conclusion, in the current study, we describe a suite of tools for NEAT1_2/paraspeckle research and various drug discovery activities, including tool molecule identification, deconvolution of HTS results or hit-to-lead optimisation activities.

## DATA AVAILABILITY

All relevant data have been included in this manuscript, and any additional data pertinent to this study are available from the corresponding author upon reasonable request.

## Supplementary Material

gkac771_Supplemental_Files

## Notes

Present address: Haiyan An, Institute of Life Science, Medical School, Swansea University, Swansea SA2 8QA, United Kingdom.

Present address: Olivera Grubisha, UCB Pharma, Slough SL1 3WE, United Kingdom.
