# Supplementary material for: A toolkit for the identification of NEAT1_2/paraspeckle modulators
Source: Nucleic Acids Res. 2022 Sep 13;50(20):e119. doi: 10.1093/nar/gkac771 (PMC9723620; doi:10.1093/nar/gkac771)
Supplement: gkac771_Supplemental_Files [file gkac771_supplemental_files.zip › Supplementary data.pdf]

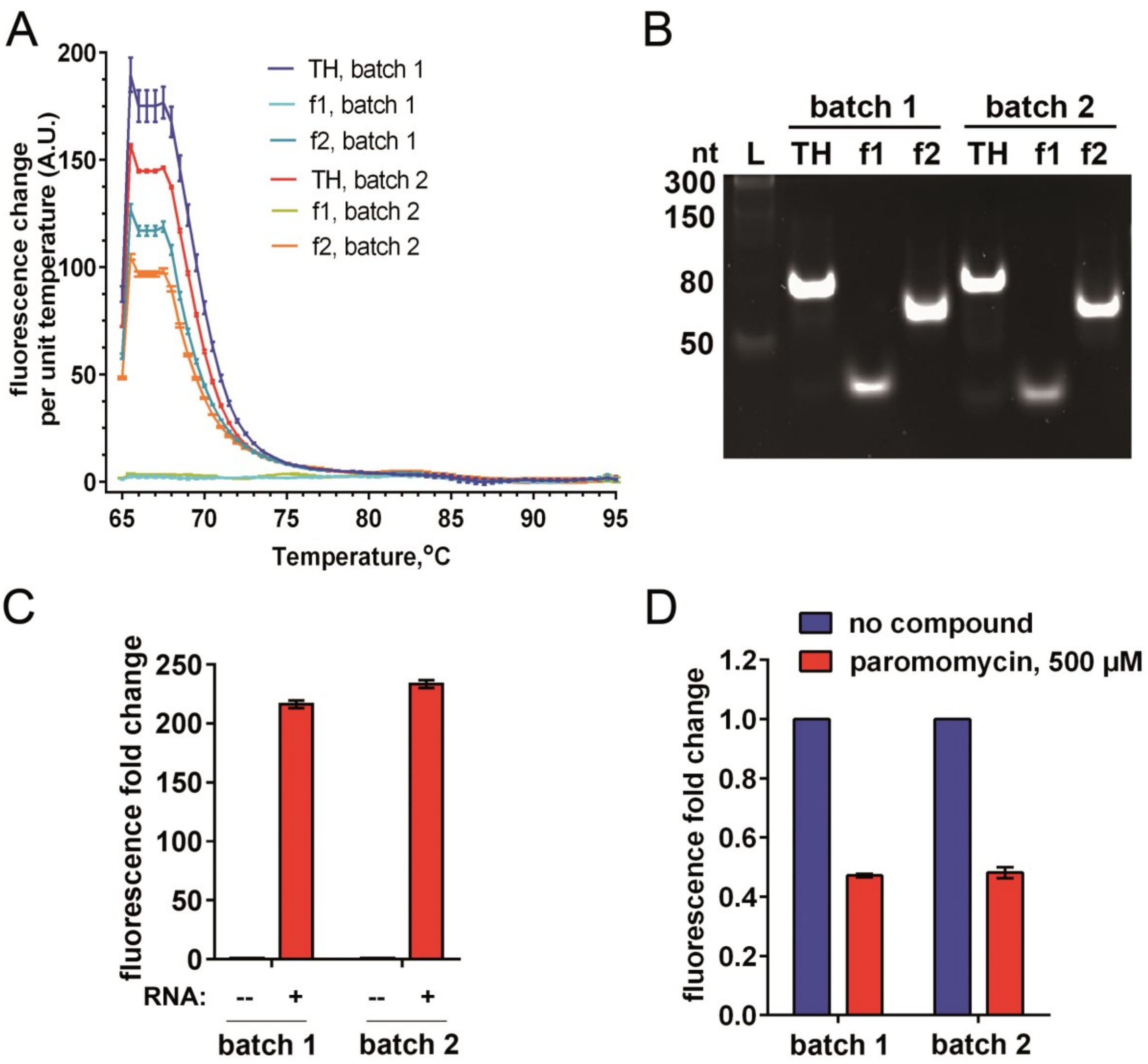

Figure S1. Limited batch-to-batch variability for commercial RNA oligonucleotides used for NEAT1_2 TH complex assembly.

(A) Thermal melting analysis for NEAT1_2 TH and RNA oligonucleotides from two independent synthesis batches.

(B) Native PAGE of NEAT1_2 TH assembled using RNA oligonucleotides from two independent synthesis batches. Representative gel is shown.

(C,D) FID assay results for NEAT1_2 TH reconstituted from RNA oligonucleotides from two independent synthesis batches. Fluorescence fold increase for TO-PRO upon RNA binding (C) and displacement by the positive assay control (D) are shown. Final RNA concentration was 0.5 µM.

The analysis was carried out in duplicates in A and D and in 5 replicates in C.

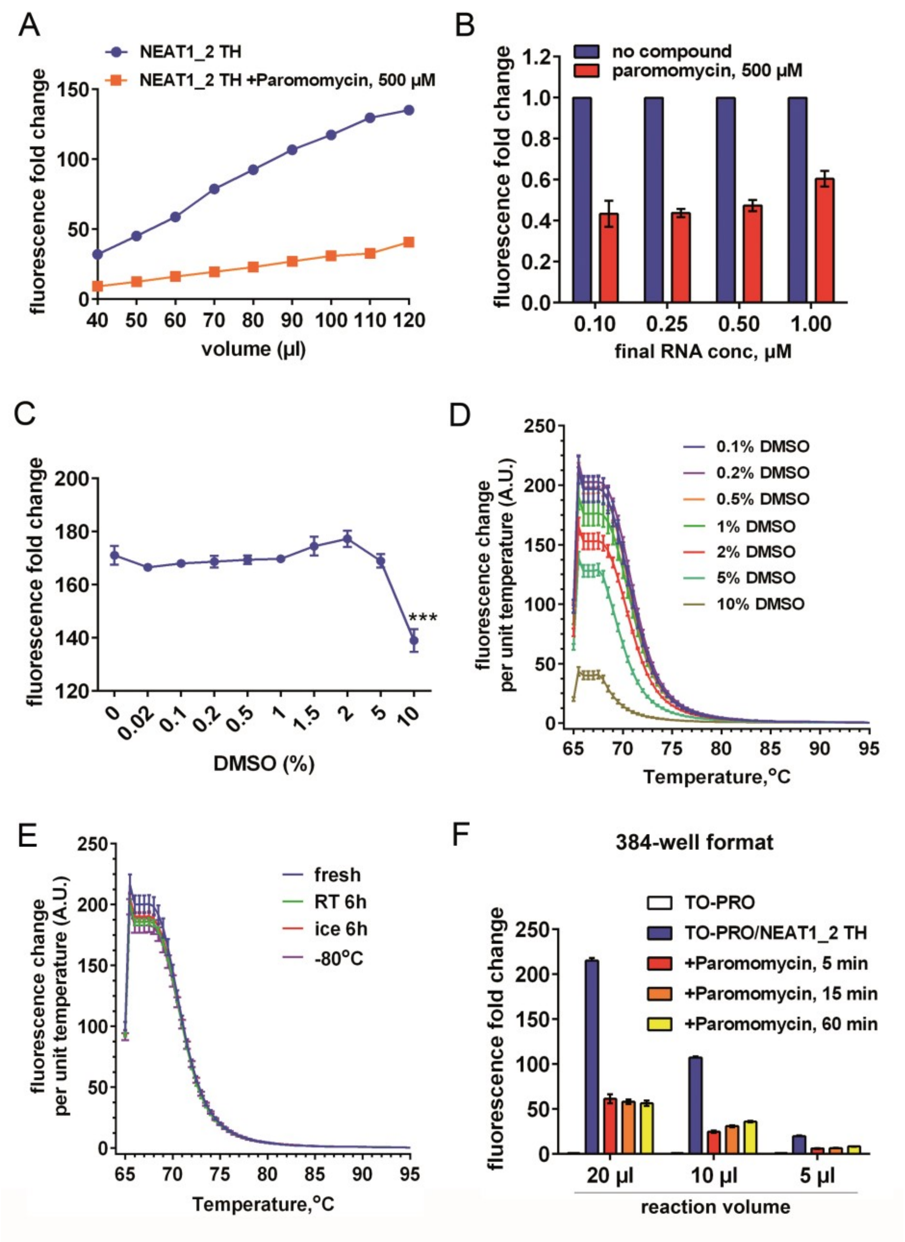

Figure S2. Optimisation of FID assay for NEAT1_2 TH RNA substrate.

(A) The effect of reaction volume on FID assay performance.

(B) Determining a minimal NEAT1_2 TH concentration required for the adequate performance of FID assay.

(C) DMSO tolerance of the NEAT1_2 TH FID assay. ***$p<0.001$ (Student's *t* test).

(D) The effect of DMSO on NEAT1_2 TH complex integrity as determined by thermal melting. DMSO was added to the preformed TH complex immediately before melting analysis.

(E) Stability of NEAT1_2 TH complex at room temperature (RT), on ice and after a freeze-thaw cycle ('-80ºC-), as compared to a freshly prepared complex.

(F) Determining a minimal reaction volume for NEAT1_2 TH FID assay in a 384-well format.

Final concentration of NEAT1_2 TH RNA was 0.5 µM in all panels, except B. Experiment was carried out in duplicates in A; in triplicates in B,D,E; in 5 replicates in C; and in 4 replicates in F.

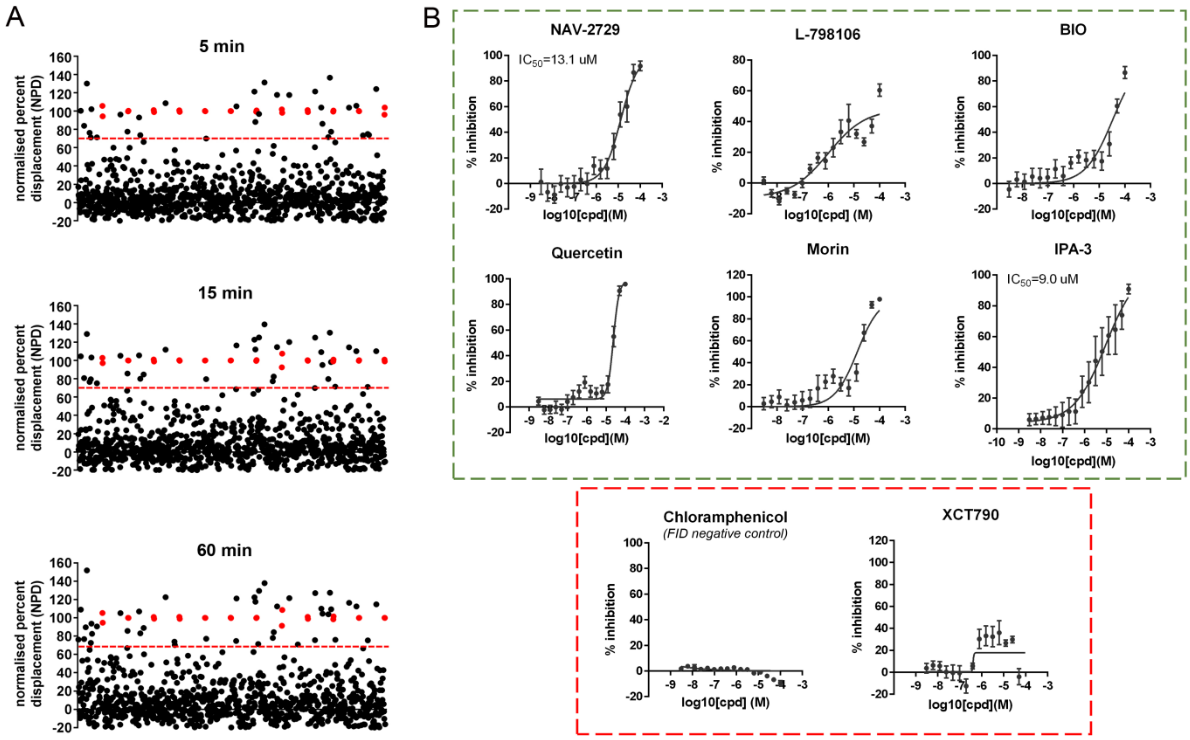

Figure S3. Pilot small molecule library screen using NEAT1_2 TH FID assay: plots for the individual time-points and dose-response curves for selected hits.

(A) Normalised percent displacement (NPD) value for each of the LOPAC®1280 library compounds after a 5-min, 15-min and 60-min incubation. Red dots correspond to the positive control (paromomycin). Red line marks the threshold used to identify hits (70% NPD).

(B) Dose-response for a panel of LOPAC®1280 hits (also see Figure 3). Chloramphenicol was the FID assay negative control. Note that XCT790 did not show a concentration response and that IPA-3 response was highly variable between replicates. IC_{50} is indicated where it was possible to reliably calculate it. N=3 for all concentrations.

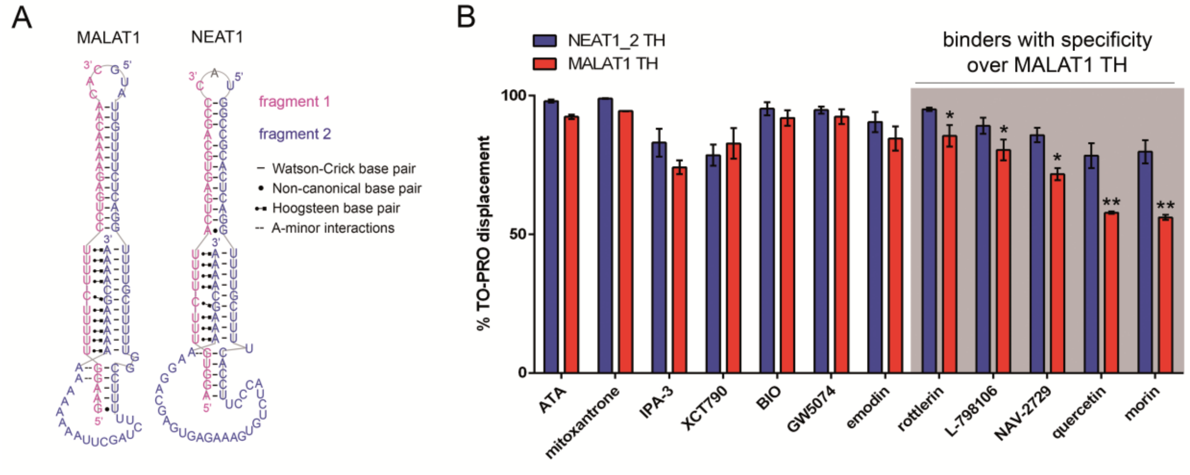

Figure S4. Selectivity of LOPAC®1280 hits.

(A) Schematic of bipartite NEAT1_2 and MALAT1 TH structures reconstituted *in vitro.*

(B) Comparison of dye displacement from NEAT1_2 TH and MALAT1 TH by the hits taken into validation studies. Binders displaying better displacement from NEAT1_2 TH are shadowed. N=3, *p<0.05, **p<0.01 (Mann-Whitney *U* test).

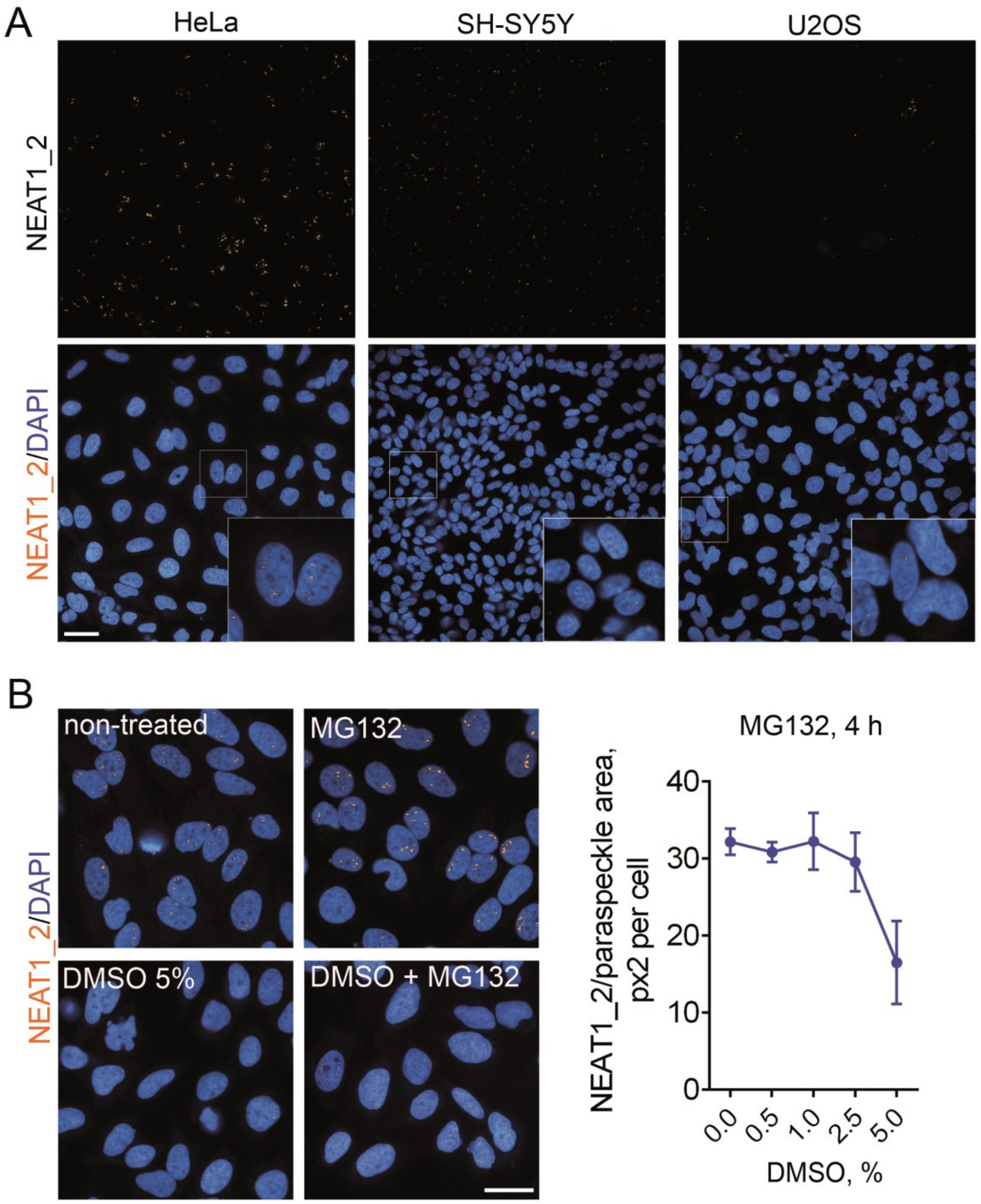

Figure S5. ParaQuant assay development: cell line variability and the effect of high DMSO.

(A) Representative images of fields taken at 40x magnification for NEAT1_2/paraspeckles and merged images with nuclei for HeLa, SH-SY5Y and U2OS cells, used for automated analyses in this study (maximum projections from three planes are shown). Note that typically between 50 and 300 cells are captured per field of view depending on the cell size and plating density. Scale bar, 20 µm.

(B) Pre-treatment with 5.0% DMSO prevents MG132-induced NEAT1_2/paraspeckle accumulation in HeLa cells. Representative images and quantification are shown. Cells were pre-treated with 5.0% DMSO for 7 h and subsequently treated with MG132 for 4 h. N=3. Scale bar, 20 µm.

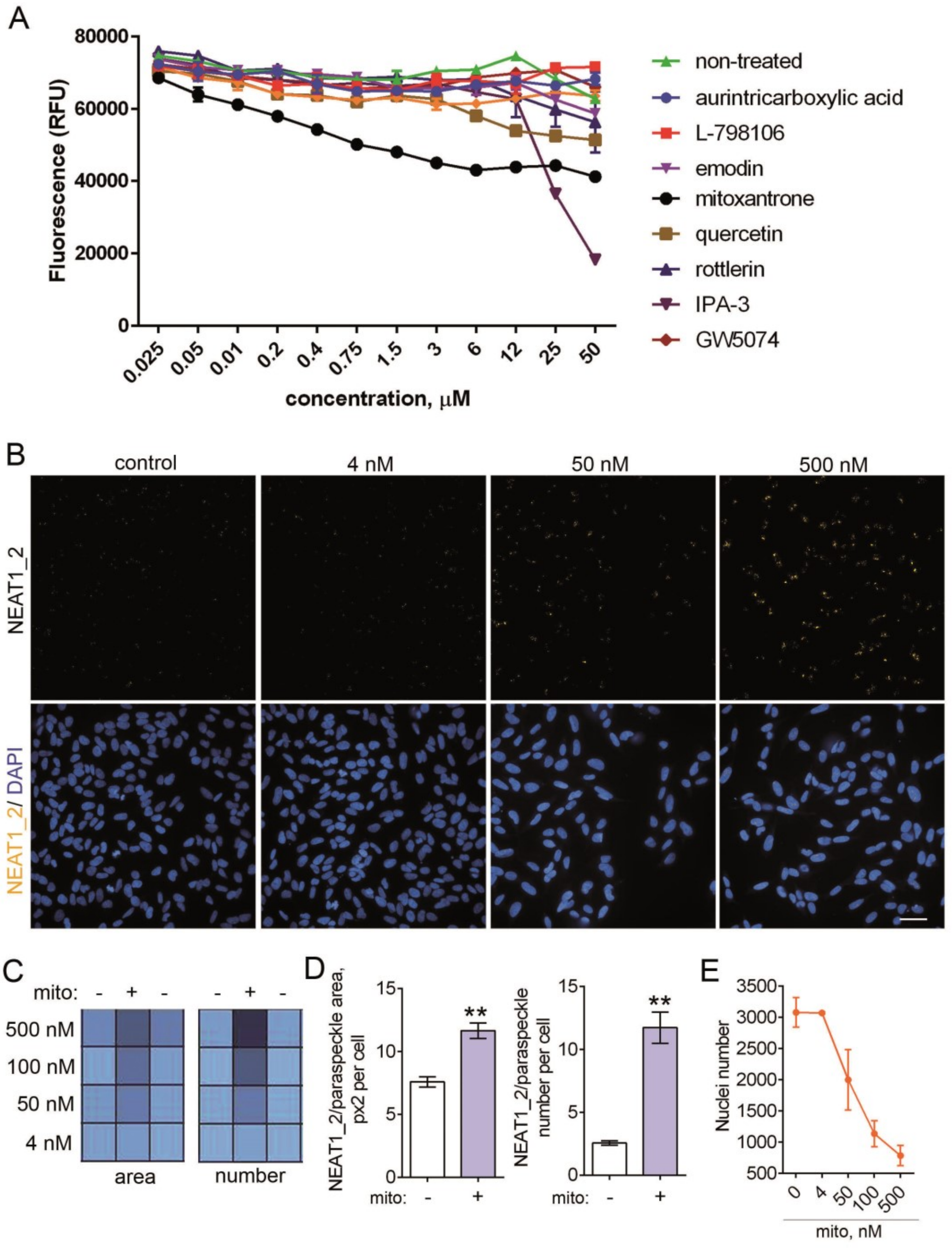

Figure S6. Cellular toxicity of LOPAC®1280 hits and analysis of mitoxantrone effect on NEAT1_2/paraspeckles.

(A) Concentration-dependent toxicity for selected LOPAC®1280 hits as measured by a resazurin-based assay (CellTiter-Blue®). SH-SY5Y cells were analysed after a 24-h treatment with the indicated concentration.

(B-D) Effect of mitoxantrone on NEAT1_2/paraspeckles in SH-SY5Y cells. Cells were treated with the indicated concentration of mitoxantrone for 24 h and analysed by ParaQuant. Representative images (B), heatmaps of an assay plate (C) and quantification (for 100 nM final concentration, D) are shown. N=4, **p<0.01 (Mann-Whitney *U* test). Mito, mitoxtantrone. Scale bar, 50 µm.

(E) Concentration-dependent toxicity of mitoxantrone in SH-SY5Y cells as measured by nuclei count recorded in ParaQuant assay. Nuclei number per 10 fields of view was plotted.

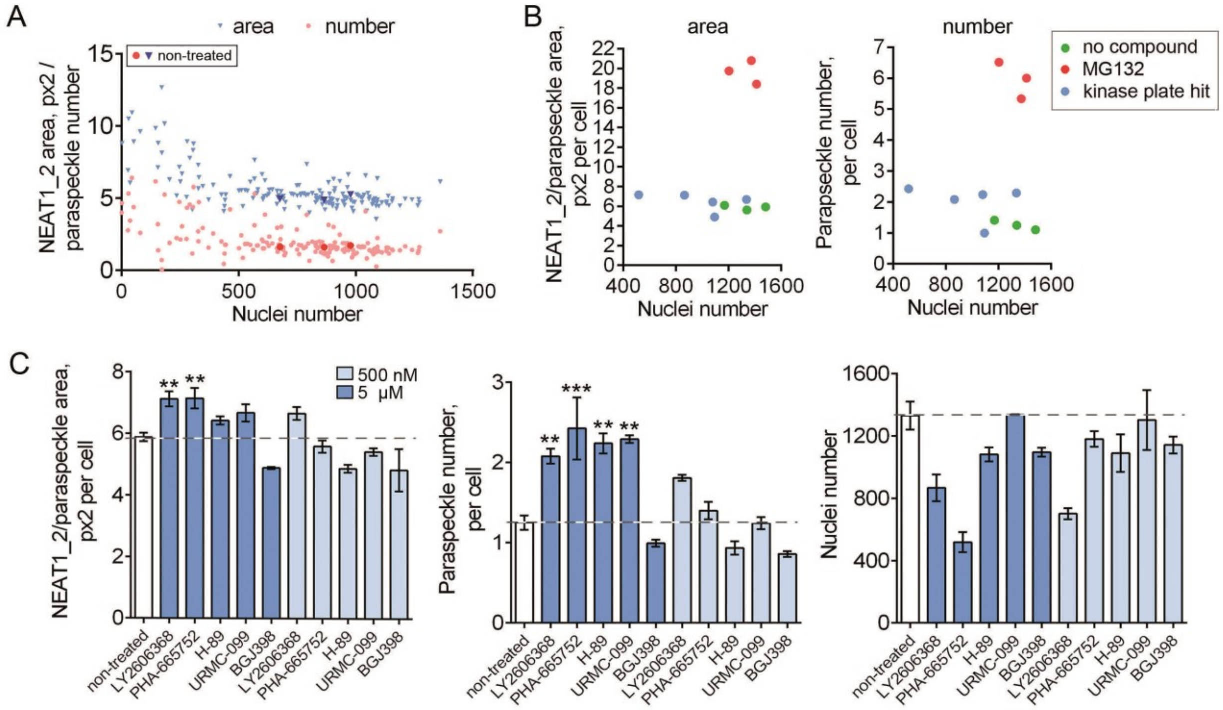

Figure S7. Validation of ParaQuant as a primary screening assay (SH-SY5Y cells) and identification of kinase inhibitors – positive paraspeckle modulators.

(A) Correlation plot for the paraspeckle readouts (area and number) combined, *vs.* nuclei number for the Cayman Chemical Kinase Library screen. Nuclei count for 10 fields of view was plotted.

(B) Correlation plots for the paraspeckle readouts (area and number) *vs.* nuclei number for the kinase inhibitor hits taken into retest (“zoom-in” from A).

(C) Retest results for the kinase inhibitor hits. Compounds were tested at 5 μM and 500 nM, with a 24-h treatment. N=3, **p<0.01, ***p<0.001 (one-way ANOVA with Dunnett’s post-hoc test). Nuclei count for 10 fields of view was used.

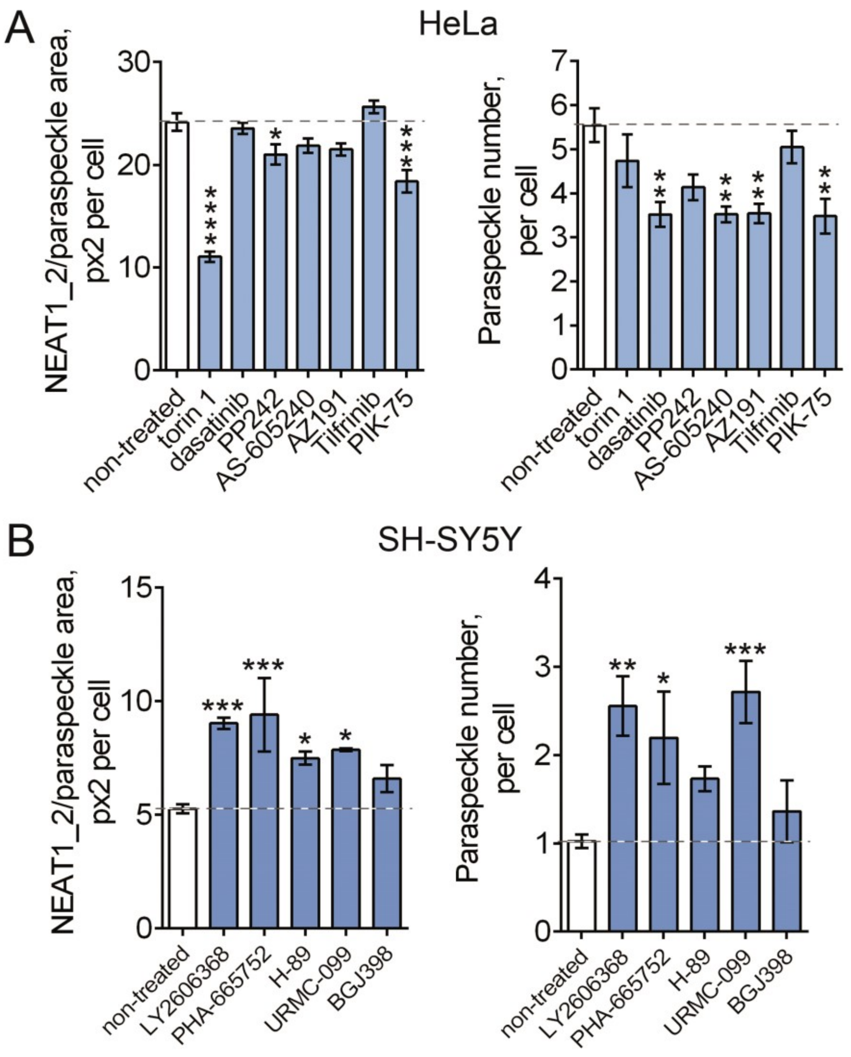

Figure S8. Retest of kinase inhibitor library hits using a miniaturised (384-well) version of ParaQuant.

(A) Kinase inhibitors - negative modulators of paraspeckles identified in HeLa cells demonstrate expected activity when tested in a 384-well format. Compounds were tested with the original assay conditions (5 μM for 24 h). N=3, *p<0.05, **p<0.01, ***p<0.001, ****p<0.0001 (one-way ANOVA with Dunnett's post-hoc test).

(B) Kinase inhibitors - positive modulators of paraspeckles identified in SH-SY5Y cells demonstrate expected activity when tested in a 384-well format. Compounds were tested with the original assay conditions (5 μM for 24 h). N=3, *p<0.05, **p<0.01, ***p<0.001 (one-way ANOVA with Dunnett's post-hoc test).

A

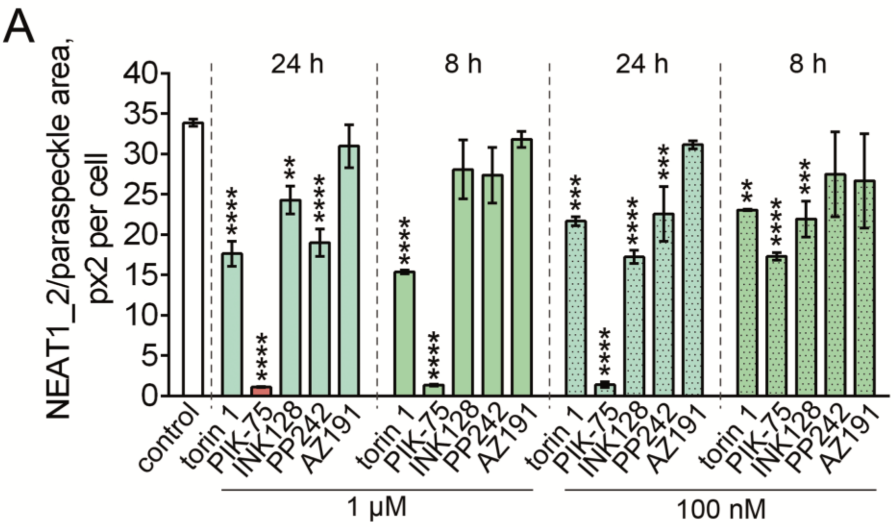

B

Kinase inhibitor	Effect on basal NEAT1_2/ paraspeckles	Target		
		PI3K	mTOR	DNA-PK
PIK-75	↓↓↓	+++	+	+++
Torin 1	↓↓↓	+	+++	+
PP242	↓↓	++	+++	++
INK128	↓↓	++	+++	-
AZ191	↓	?	++?	?
LY3023414	↓↓	+++	++	+++
PI-103	↓↓	+++	+++	+++
NU 7026	no effect	-	-	+++
Wortmannin	no effect	+++	-	+++
GSK1059615	↓	+++	+++	-
CAY10626	↓	+++	+++	-
TGX-221	no effect	+++ (p110β)	-	-
CAY10505	no effect	+++(p110γ)	-	-

+++ *low nanomolar* ++ *high nanomolar* + *low micromolar*

red - paraspeckle inhibitors *shadowed - independent compound batch tested*

Figure S9. Identification of dual PI3K/mTOR inhibitors as negative NEAT1_2/paraspeckle modulators.

(A) TORkinibs, PIK-75 and AZ191 from an independent batch/provider were tested in HeLa cells in two concentrations, for 8 and 24 h. PIK-75 was tested at 250 nM and 25 nM instead of 1 µM and 100 nM, respectively, due to toxicity. Note that PIK-75 at 250 µM for 24 h (red coloured bar) was still highly toxic, as judged by nuclei count. Compounds were analysed in duplicates. **p<0.01, ***p<0.001, ****p<0.0001 (one-way ANOVA with Dunnett's post-hoc test).

(B) Summary of compounds' inhibitory activities for the panel of compounds tested within the kinase inhibitor library and from an independent batch/provider. Compounds in white fields were tested as part of the library only.

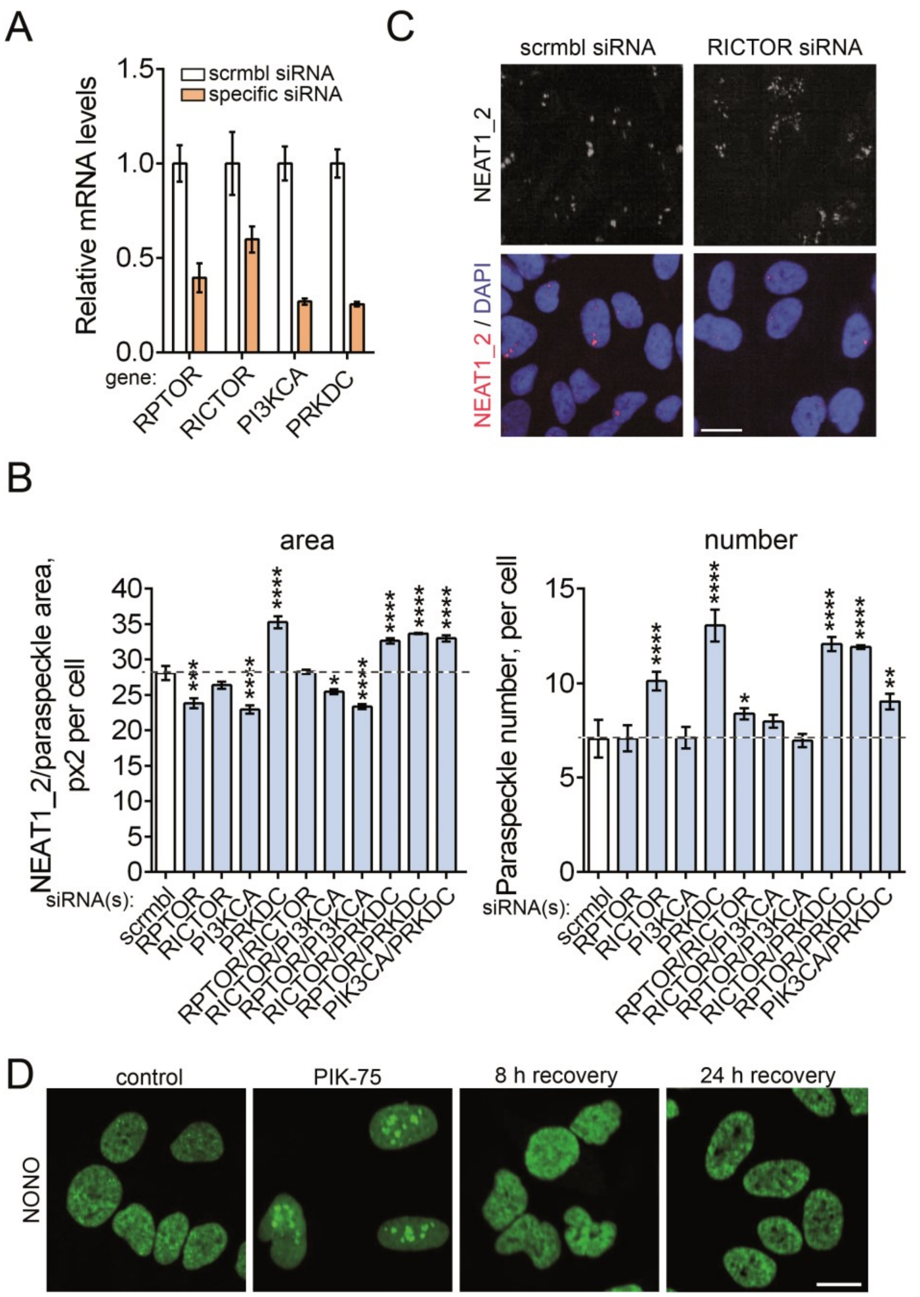

Figure S10. Manipulation of candidate pathways by siRNA knockdown confirms the involvement of PI3K/mTOR pathway in paraspeckle maintenance.

(A) Verification of gene knockdown by siRNA as by qRT-PCR. HeLa cells were analysed 48 h post-transfection, in duplicates.

(B) NEAT1_2/paraspeckle levels after siRNA knockdown of raptor, rictor, PI3KCA and PRKDC, as well as double knockdown. HeLa cells were analysed using ParaQuant setup 48 h post-transfection. N=3, *$p<0.05$, **$p<0.01$, ***$p<0.001$, ****$p<0.0001$ (one-way ANOVA with Dunnett's post-hoc test).

(C) Rictor depletion leads to smaller, dispersed paraspeckles. HeLa cells were analysed 48 h post-transfection with siRNAs. Representative images are shown. Scale bar, 20 µm.

(D) PIK-75-induced paraspeckle protein redistribution in the nucleus is reversible. HeLa cells were treated by 25 nM PIK-75 for 24 h and left to recover for 8 or 24 h. Representative images for NONO are shown. Scale bar, 10 µm.

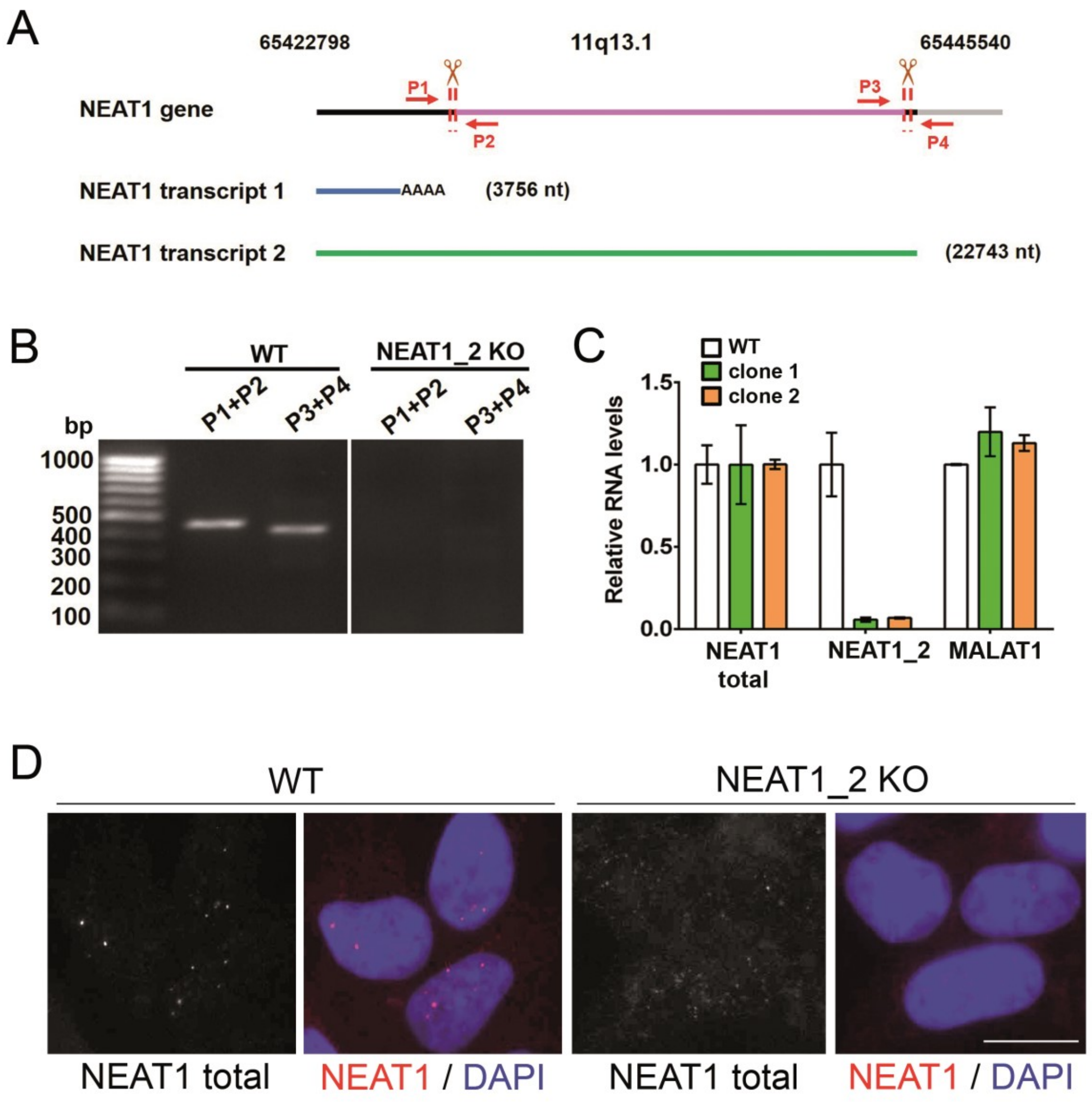

Figure S11. Generation of NEAT1_2 knockout (KO) SH-SY5Y cells.

(A) CRISPR/Cas9 editing of the locus: position of sgRNAs and screening primers (P1-4).

(B) A positive clone verified by PCR with two different primer combinations.

(C) Verification of NEAT1_2 loss of expression and normal expression of NEAT1_1 isoform, as well as MALAT1, in two clones of NEAT1_2 KO cells using qRT-PCR. N=3.

(D) Loss of larger foci (paraspeckles) and accumulation of NEAT1_1-positive “microspeckles” in a NEAT1_2 KO clone, as revealed by RNA-FISH with a total NEAT1 (5’-end specific) probe. Scale bar, 10 μm.

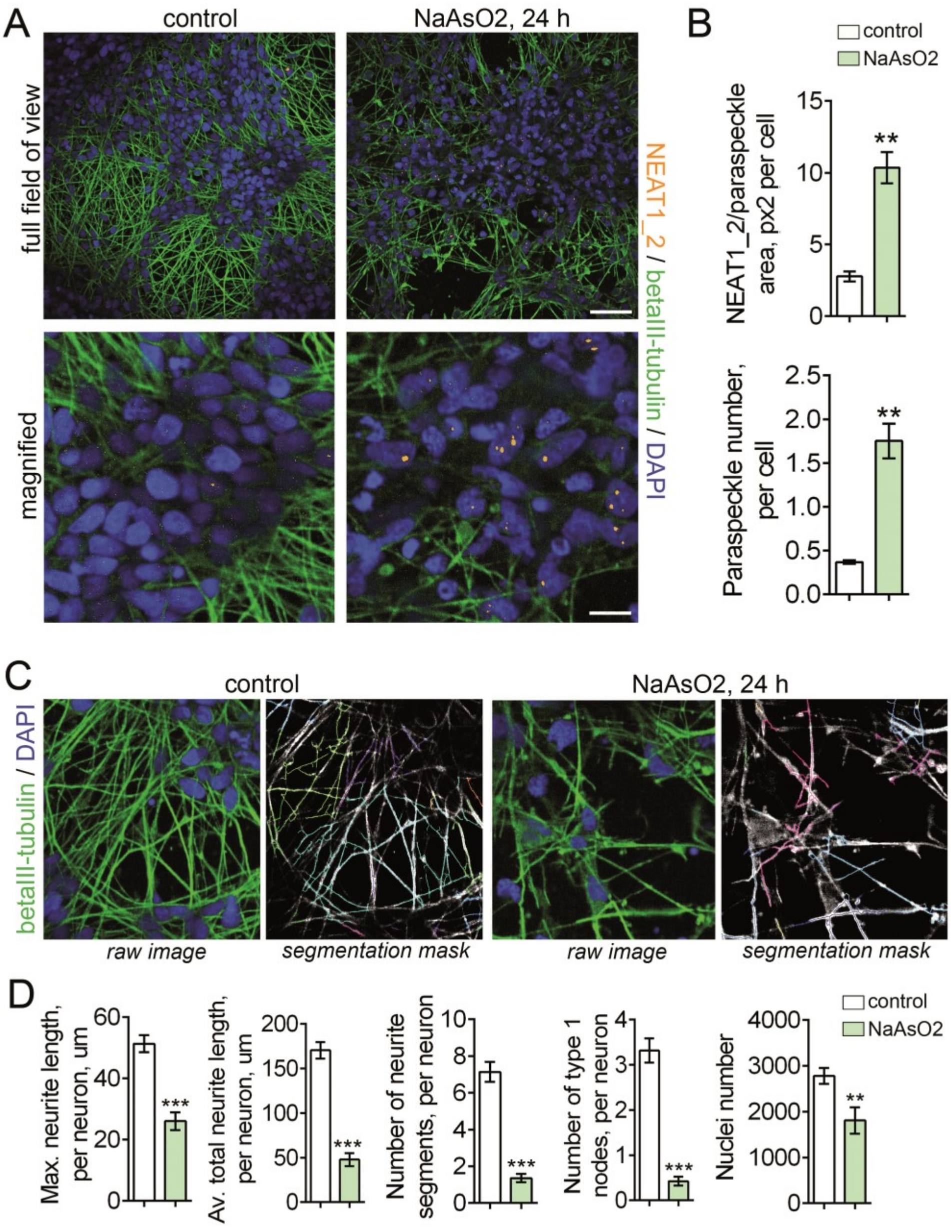

Figure S12. Multiplexing and analysis of additional cellular phenotypes with the ParaQuant assay: human motor neurons.

(A) Visualisation of NEAT1_2/paraspeckles and the neurite network in human ES-derived motor neurons. Day 40 human motor neurons were subjected to $NaAsO_2$ stress for 1 h and left to recover for 24 h. Representative images are shown. Neurite network was visualised with Alexa Fluor®488-conjugated anti-betaIII-tubulin antibody. Scale bars, 100 and 20 µm for top and bottom panels respectively.

(B) NEAT1_2/paraspeckle quantification in stressed neurons. Neurons were treated as described above. N=4, **p<0.01 (Mann-Whitney *U* test).

(C) Representative segmentation masks for neurites used in automated analysis of the neurite network integrity.

(D) Quantitative analysis of the neurite network in stressed neurons. Neurons were treated as described above. N=4, **p<0.01, ***p<0.001 (Mann-Whitney *U* test). Nuclei count for 10 fields of view is shown.

Table S1. LOPAC®1280 library hits (available as Excel file)

Table S2. Kinetic parameters for the compounds binding to NEAT1_2 TH and individual RNA oligonucleotides (fragments) determined by GCI-waveRAPID method.

**Compound**	**Substrate**	**ka M-1s-1**	**ka error%**	**kd s-1**	**kd error%**	**K_D µM**	**Rmax pg/mm2**	**Sqrt(Chi2) pg/mm2**
**GW5074**	NEAT1_2 TH	1.09E+05	7.20E+00	3.73E-01	4.12E+00	3.4	9.23	0.16
	Fragment 1	6.18E+04	2.37E+01	8.90E-01	8.58E+00	14.4	8.30	0.13
	Fragment 2	-	-	-	-	Outside measurable limits	-	0.15
**Emodin**	NEAT1_2 TH	1.92E+05	1.27E+01	4.38E-01	8.88E+00	2.284	1.95	0.11
	Fragment 1	-	-	-	-	Outside measurable limits	-	0.20
	Fragment 2	-	-	-	-	Outside measurable limits	-	0.18
**Morin**	NEAT1_2 TH	7.85E+04	1.93E+01	1.83E-02	2.74E+01	0.232	6.56	0.22
	Fragment 1	5.35E+04	2.78E+01	3.98E-02	3.46E+01	0.745	3.81	0.20
	Fragment 2	1.47E+04	4.97E+01	3.36E-02	3.06E+01	2.289	4.82	0.14
**Paromomycin**	NEAT1_2 TH	32352.35	9.46	0.26	2.96	7.90	141.46	0.39
	Fragment 1	17272.98	14.83	0.23	3.02	13.04	149.82	0.35
	Fragment 2	11970.44	37.01	0.20	6.00	16.71	143.17	0.39
**L798106**	NEAT1_2 TH	1.02E+05	5.06E+00	2.41E-01	2.84E+00	2.368	10.21	0.14
	Fragment 1	-	-	-	-	Outside measurable limits	-	0.22
	Fragment 2	1.26E+05	2.27E+01	7.48E-01	1.39E+01	5.918	2.51	0.12

**Rottlerin**	NEAT1_2 TH	-	-	-	-	Outside measurable limits	-	0.18
	Fragment 1	6.33E+03	1.47E+01	2.24E-02	4.41E+00	3.542	50.16	0.15
	Fragment 2	5.62E+03	1.32E+01	1.90E-02	3.64E+00	3.386	25.94	0.10
**NAV-2729**	NEAT1_2 TH	3.29E+03	4.48E+01	6.45E-02	6.87E+00	19.619	17.54	0.11
	Fragment 1	4.34E+03	4.67E+01	4.93E-02	9.24E+00	11.355	15.26	0.11
	Fragment 2	8.04E+03	3.27E+01	5.04E-02	1.17E+01	6.268	7.27	0.09
**Quercetin**	NEAT1_2 TH	8.98E+04	2.24E+01	2.57E-02	2.84E+01	0.286	7.47	0.24
	Fragment 1	4.45E+04	2.55E+01	3.49E-02	2.95E+01	0.784	5.07	0.21
	Fragment 2	5.61E+03	6.84E+01	4.64E-02	1.73E+01	8.256	11.92	0.12

Table S3. Results of the Cayman Chemical Kinase Library screens in HeLa and SH-SY5Y cells (available as Excel file).
